# Accurate recapture identification for genetic mark–recapture studies with error-tolerant likelihood-based match calling and sample clustering

**DOI:** 10.1098/rsos.160457

**Published:** 2016-12-21

**Authors:** Suresh A. Sethi, Daniel Linden, John Wenburg, Cara Lewis, Patrick Lemons, Angela Fuller, Matthew P. Hare

**Affiliations:** 1 US Geological Survey, New York Cooperative Fish and Wildlife Research Unit, Cornell University, Ithaca, NY 14853, USA; 2 New York Cooperative Fish and Wildlife Research Unit, Department of Natural Resources, Cornell University, Ithaca, NY, USA; 3 Conservation Genetics Laboratory, US Fish and Wildlife Service, Anchorage, AK 99503, USA; 4 Marine Mammals Management, US Fish and Wildlife Service, Anchorage, AK 99503, USA; 5 Department of Natural Resources, Cornell University, Ithaca, NY, USA

**Keywords:** sample matching, genotyping error, inference, non-invasive, capture–recapture

## Abstract

Error-tolerant likelihood-based match calling presents a promising technique to accurately identify recapture events in genetic mark–recapture studies by combining probabilities of latent genotypes and probabilities of observed genotypes, which may contain genotyping errors. Combined with clustering algorithms to group samples into sets of recaptures based upon pairwise match calls, these tools can be used to reconstruct accurate capture histories for mark–recapture modelling. Here, we assess the performance of a recently introduced error-tolerant likelihood-based match-calling model and sample clustering algorithm for genetic mark–recapture studies. We assessed both biallelic (i.e. single nucleotide polymorphisms; SNP) and multiallelic (i.e. microsatellite; MSAT) markers using a combination of simulation analyses and case study data on Pacific walrus (*Odobenus rosmarus divergens*) and fishers (*Pekania pennanti*). A novel two-stage clustering approach is demonstrated for genetic mark–recapture applications. First, repeat captures within a sampling occasion are identified. Subsequently, recaptures across sampling occasions are identified. The likelihood-based matching protocol performed well in simulation trials, demonstrating utility for use in a wide range of genetic mark–recapture studies. Moderately sized SNP (64+) and MSAT (10–15) panels produced accurate match calls for recaptures and accurate non-match calls for samples from closely related individuals in the face of low to moderate genotyping error. Furthermore, matching performance remained stable or increased as the number of genetic markers increased, genotyping error notwithstanding.

## 1. Introduction

While the number and breadth of genetic mark–recapture applications is increasing, challenges in constructing capture histories from multilocus genotypes remain [1]. Genotyping errors are common with low-quality, low-quantity DNA samples from non-invasive genetic mark–recapture studies [2] and can result in missed recapture events which inflate mark–recapture abundance estimates [3]. Genetic marker panels with low information content can produce false recapture events, potentially biasing abundance estimates low [4]. The discriminating power of genetic marker panels can be increased with additional loci (with additional development and genotyping costs); however, larger panels increase the probability of additional genotyping error events. A suite of approaches have been suggested to deal with genotyping error in constructing capture histories for genetic mark–recapture studies. Broadly, these fall into two categories: (i) remove genotyping errors from the data and (ii) develop sample matching protocols that are robust to genotyping errors. Considerable guidance exists on removing genotyping errors from data, including careful sample preparation [5], replicate genotyping through a multitubes approach [6–8] and filtering out low-quality genotype calls for exclusion or additional genotyping [9].

More recently, error-tolerant matching approaches, which can accommodate low levels of genotyping error while producing accurate match calls, have shown promise. Two analogous error-tolerant likelihood-based sample matching protocols were introduced by Wang [10–12] and Kalinowski *et al*. [13]. Both approaches use the same structure in combining probabilities of obtaining a pair of true underlying, or latent, genotypes given population allele frequencies and hypotheses about the relationship state between the two samples (e.g. samples from full siblings or unrelated individuals), coupled with the probability of observing the sample genotypes given a genotyping error model and genotyping error rates. The two approaches differ in the manner in which genotyping error is modelled and in the clustering algorithms implemented to group samples into putative same-individual sets.

Likelihood-based matching has several benefits. First, the approach is based upon sound probability theory describing the frequency of genotypes in a population (e.g. [14]). Second, the approach allows for statistical inference about match calls in the presence of genotyping errors. Finally, match calling when comparing pairs of observed genotypes is objective and based upon the strength of evidence metrics, whereas some other non-likelihood-based approaches use heuristic matching criteria which require tuning (e.g. [3,15]). The adoption of error-tolerant likelihood-based match calling and sample clustering approaches, however, has been slow in genetic mark–recapture applications, probably owing to their high complexity and high computation requirements (see below). While earlier work laid a foundation for error-tolerant likelihood-based match calling and sample clustering [10–13], additional practical guidance on implementing the approach for genetic mark–recapture studies and assessing what are requirements for genetic marker panel size and quality (allelic richness, genotyping error levels) necessary to accurately reconstruct capture histories for mark–recapture modelling may facilitate broader use of these tools.

Kalinowski *et al*. [13] introduced and explored the performance of an error-tolerant likelihood-based match calling model specified with a detailed genotyping error model for microsatellite (MSAT) genetic markers. In addition to likelihood-based sample matching, they introduced a likelihood model for estimating parameters of the genotyping error model from sample data. Simulation results in this work explored the combined performance of the match calling model under the specified MSAT error model, the genotyping error estimation model and a proposed sample clustering algorithm. Wang ([12], building from Wang [10,11]), explored performance of an analogous error-tolerant likelihood-based match calling model as that of Kalinowski *et al*. [13], specifying a different MSAT-specific genotyping error model than from [13] and asserting genotyping error rates as opposed to estimating them from sample data. Wang [12] implemented a sample clustering algorithm which first identifies sibling clusters and then identifies duplicate multilocus genotypes, i.e. recapture events, within sibling clusters; this routine was made available in the software package COLONY (written by J. Wang, available at www.zsl.org/science/software/colony; therein, refer to the option to identify ‘clones’ from multilocus genotype samples for the purposes of identifying recapture events from genetic mark–recapture samples). Performance of the combined match calling model and clustering algorithms was assessed under a suite of simulations examining the effect of marker panel size and quality (allelic richness), error rates and misspecification of error rates. These earlier works provided the groundwork for implementing error-tolerant likelihood-based match calling and sample clustering for genetic mark–recapture studies; however, testing performance results were presented combining multiple features of the protocols and for a limited set of simulation scenarios. For example, Kalinowski *et al*. [13] and Wang [12] presented the combined performance of both the match calling model and the clustering algorithms with predefined multilocus genotypes sample sizes, which combined mixes of related and unrelated individuals. Furthermore, the accuracy of reconstructing recaptures was assessed by evaluating the combined match and non-match call performance for simulated sample sets.

In this article, we expand upon these earlier efforts [10–13] to provide guidance on using error-tolerant likelihood-based sample clustering algorithms for designing and analysing genetic mark–recapture studies. A combination of simulation trials and case studies on Pacific walrus (*Odobenus rosmarus divergens*) and fishers (*Pekania pennanti*) are analysed with the objective of providing guidance on the number of markers, allelic richness and genotyping error rates which are acceptable in achieving accurate recapture histories for genetic mark–recapture studies. We focus simulation testing performance at the level of the error-tolerant likelihood-based match calling model. Results for both MSAT and single nucleotide polymorphism (SNP) genotypes are assessed for unrelated or full-sibling relationship states in isolation, and for match and non-match call accuracy separately. A distinct genotyping error model is proposed for SNP genotypes, and we explore sensitivity of the match calling model to errors in model input parameters, including misspecification of allele frequencies and genotyping error rates. Second, we develop a two-stage genotype clustering protocol designed to accommodate typical sampling scenarios from genetic mark–recapture studies, whereby repeated captures may occur within sampling occasions. Combining match calling performance testing with the proposed sample-clustering algorithms, analysts can use results herein to anticipate recapture reconstruction accuracy for genetic mark–recapture sampling scenarios specific to their study of interest. Finally, we provide example code written in the R statistical programming environment [16] to implement the error-tolerant likelihood-based match calling and two-stage sample clustering protocol. Simulations indicate that the error-tolerant likelihood-based match calling model can accommodate moderate genotyping error while still making accurate match calls with reasonably sized SNP and MSAT panels.

## 2. Material and methods

All simulation data generation, performance testing and case study recapture reconstruction analyses were conducted with custom scripts written in R.

### 2.1. Error-tolerant likelihood-based match calling model and sample clustering algorithm

The error-tolerant likelihood-based match calling approach analysed here follows the matching probability model presented in Wang [10–12] and Kalinowski *et al*. [13]; however, the sample clustering algorithm we implement to group samples into putative same-individual sets differs from both the above approaches, and we implement separate genotyping error models for SNP and MSAT markers. A brief description of the approach is outlined here; complete equations and sample clustering algorithm detail are provided in the electronic supplementary material.

The match calling model operates on pairwise comparisons of multilocus genotypes and uses a strength-of-evidence approach to determine a match. The match calling model calculates the likelihood of observing a pair of multilocus genotypes given a hypothesis about the relationship state from which the samples were derived, i.e. samples came from the same individual in which case the pair is a match, or the samples came from two different individuals. The model for the probability of observing a given multilocus genotype incorporates both the probability of a latent, true underlying genotype, calculated from population allele frequencies, as well as the probability of observing the sample genotypes given a genotyping error model. To discriminate recapture events from unique specimens, a clustering algorithm is specified to examine pairwise comparisons of samples, assess whether a match call is supported using the match calling model and group samples into same-individual sets as warranted.

The framework for identifying recaptures using error-tolerant likelihood-based match calling and sample clustering encompasses four distinct components. First, a model for genotyping error is defined and used to calculate the probability of observing a diploid genotype at locus *j*, *g_j_*, given a proposed true latent genotype, *k_j_*. We implemented an MSAT genotyping error model following Wang [10], which specifies two types of error: allelic dropouts and mistypes (also referred to as ‘false alleles’). Allelic dropouts occur when heterozygote genotypes are read as homozygotes, typically attributed to greater amplification efficiency of a smaller allele over a larger allele during polymerase chain reaction (PCR). Mistype errors have several possible causes and can involve a miscalled allele in either homozygote or heterozygote genotypes. Biallelic SNPs consist of a single base pair polymorphism and we implemented a simple generic genotyping error model whereby alleles are either called correctly or not in a binomial probability framework (electronic supplementary material, S1).

Second, a model is proposed for the probability of observing a pair of latent multilocus genotypes (unlinked loci, codominant alleles, random mating) given a proposed relationship state and set of population allele frequencies, following standard formulae for relatedness analysis (e.g. [14]).

Third, the joint probability of observing the pair of sample multilocus genotypes, *G*_1_ and *G*_2_, incorporating both population allele frequencies and genotyping error are used to calculate the likelihood of a hypothesized relationship state, *R*, (e.g. R∈{SI, U, FS, PO}, where U = samples from unrelated individuals, FS = samples from a pair of full siblings, PO = samples from a parent offspring pair and SI = a pair of samples from the same individual), *L*(*R*(*G*_1_, *G*_2_)). The strength of evidence for the SI relationship state, i.e. a match call, is assessed by calculating the ratio of the likelihood of the SI relationship state being true against the maximum-likelihood non-match relationship state:

$$\Lambda =\frac{L(R(G_{1},G_{2})=\text{SI})}{\text{max}\{L(R(G_{1},G_{2})=U),L(R(G_{1},G_{2})=\text{FS}),L(R(G_{1},G_{2})=\text{PO})\}}.$$

If Λ>1.0, then a match call is made; else the samples are inferred to have come from different individuals. We specified a match calling probability model as presented by Kalinowski *et al*. [13] which accommodates multiple recaptures of an individual within a set of samples, in which case likelihoods are evaluated for pairs of compared sets of genotypes (electronic supplementary material, S1).

Finally, a sample clustering algorithm is proposed which groups samples into sets with genotypes from the same individual. We implement a two-stage sample clustering approach to identify recapture events with the error-tolerant likelihood-based match calling model for genetic mark–recapture studies. In stage-one clustering, an algorithm is implemented to identify repeated captures within a single sampling occasion. Given a list of size *n*_1_ genotype sets from a sampling occasion, $S_{1}=(G_{1},\;\ldots ,G_{n_{1}})$ ordered with an indexing sequence of *z* = (1, … , *n*_1_):

Step 1: Define *S* = *S*_1_ with each sample in *S*_1_ as a singleton set.
Step 2: Compare the first genotype set in the list, *G*_1_, against all other genotype sets in *S*, *G_z_* for *z* > 1, and combine sets into *G*_1_ as a match when Λ>1.0.
Step 3: Compare the next genotype in sequence, e.g. *G*_2_, against all other remaining sets in *S*, *G_z_* for *z* > 2, combining sets as a match when Λ>1.0, and repeat until the last genotype set in sequence is reached, generating an updated set of genotypes, $\tilde{S}=({\tilde{G}}_{1},\ldots ,{\tilde{G}}_{{\tilde{n}}_{1}})$, where ${\tilde{n}}_{1}\leq n_{1}$.
Step 4: Repeat Steps 2–3 with $S=\tilde{S}$; if no set memberships change, stop; else repeat this step.

After completion of this algorithm, sets within $\tilde{S}$ with two or more genotypes indicate repeated captures within a sampling occasion and can be condensed into a single unique multilocus genotype (possibly reconstructing consensus genotypes from repeated captures of the same individual). Stage-one clustering would be implemented for each sampling occasion in the mark–recapture study. Subsequently, a second-stage algorithm is implemented to identify recaptures across lists of unique individuals for each sampling occasion, ${\tilde{S}}_{1}=(G_{1,1},\ldots ,\;G_{1,{\tilde{n}}_{1}})$ and ${\tilde{S}}_{2}=(G_{2,1},\ldots ,\;G_{2,{\tilde{n}}_{2}})$:

Step 1: Compare the first genotype set in sequence in ${\tilde{S}}_{1}$ against all genotype sets in ${\tilde{S}}_{2}$ and combine sets as a match, when Λ>1.0. Sets from ${\tilde{S}}_{2}$ which are combined into a given set in ${\tilde{S}}_{1}$ are removed from ${\tilde{S}}_{2}$.
Step 2: Compare the next genotype in sequence in ${\tilde{S}}_{1}$ against all remaining sets in ${\tilde{S}}_{2}$ combining sets as a match when Λ>1.0 as in Step 1, and repeat until the last genotype set in sequence in ${\tilde{S}}_{1}$ is compared against all remaining sets in ${\tilde{S}}_{2}$ generating updated sets of genotypes, $S_{1}^{*}$ and $S_{2}^{*}$.

After this second-stage clustering, sets in $S_{1}^{*}$ with two genotypes indicate recapture events; singleton sets in $S_{1}^{*}$ and $S_{2}^{*}$ represent unique individuals not recaptured across the pair of compared sampling occasions. Note that this version of the clustering algorithm assumes the starting sample sets ${\tilde{S}}_{1}$ and ${\tilde{S}}_{2}$ are made up solely of unique individuals. In this case, any given individual can only be recaptured once across a pair of compared sampling occasions' lists of unique genotypes, and only a single iteration of the algorithm is necessary. Stage-two clustering would be implemented for each pairwise comparison of sampling occasions within the mark–recapture study and results ultimately translated to individual capture histories. Electronic supplementary material, S2 and S3 provide example R code to implement the error-tolerant likelihood-based match calling model and sample clustering algorithms.

### 2.2. Simulation scenarios

Artificial MSAT and SNP multilocus diploid genotypes with codominant alleles were simulated under a range of marker set sizes, allelic richness, allele frequency specifications and genotyping error rates expected to span most genetic mark–recapture scenarios (table 1; see electronic supplementary material, S2 and S3 for example R code). Locus-specific latent genotypes were generated by randomly sampling with probability equal to a set of specified allele frequencies (i.e. assuming random mating). Full-sibling pairs were simulated by first generating two parents and then randomly sampling from each parent's respective alleles to generate two progeny. Allele frequencies for MSAT genotypes were modelled as uniform across all alleles and equal to 1/*a_j_* for *a_j_* alleles at locus *j*.

**Table 1.** Simulation scenarios for performance testing the error-tolerant likelihood-based match calling model.^a^

marker scenarios	no. loci	no. alleles	allele frequencies	error rate (per locus)	allele specification	error rate specification
SNP: baseline	32, 48, 64, 80, 96, 128	2	MAF: 0.2, 0.3, 0.4	0.00, 0.01, 0.02, 0.05, 0.10, 0.25	equivalent to data generation	equivalent to data generation
SNP: misspecified error rates	48, 64	2	0.3	0.01, 0.10	equivalent to data generation	error rates underestimated by 50% and overestimated by 50%
SNP: misspecified allele frequencies	48, 64	2	0.3	0.01, 0.10	MAF underestimated by 0.1 or overestimated by 0.1	equivalent to data generation
SNP: inclusion of high-error loci	64	2	0.3	48 loci at 0.01,16 at 0.10; 48 loci at 0.10, 16 at 0.25	equivalent to data generation	equivalent to data generation
MSAT: baseline	5, 10, 15, 20	5, 10, 20	all alleles equal, frequency = 1/(no. alleles)	(ADO, FA): (0.00, 0.00), (0.01,0.01), (0.05,0.02), (0.20,0.05)	equivalent to data generation	equivalent to data generation
MSAT: misspecified error rates	10, 15	10	all alleles equal, frequency = 1/(no. alleles)	(ADO, FA): (0.05,0.02), (0.20,0.05)	equivalent to data generation	error rates underestimated by 50% and overestimated by 50%
MSAT: misspecified allele frequencies	10, 15	10	all alleles equal, frequency = 1/(no. alleles)	(ADO, FA): (0.05,0.02), (0.20,0.05)	five alleles at 0.15 and 5 alleles at 0.05	equivalent to data generation
MSAT: inclusion of high-error loci	15	10	all alleles equal, frequency = 1/(no. alleles)	(ADO, FA): 10 loci at (0.05,0.02), 5 loci at (0.20,0.05)	equivalent to data generation	equivalent to data generation

^a^Genotyping error rates are per-locus; simulations convert per-locus error rates to per-allele rates (see Material and methods). MAF, minor allele frequency; ADO, allelic dropout; FA, false allele.

‘Observed’ genotypes containing genotyping error were generated from latent genotypes following either an MSAT- or SNP-specific error model (electronic supplementary material, S1). Locus-level error rates were first converted to allele-level error rates as: $\text{allele}\_\text{rate}=1-\sqrt{1-\text{locus}\_\text{rate }}$. For MSAT genotypes, allelic dropouts, which can only occur for latent heterozygous genotypes, were simulated first, prior to any false allele error. Under the Wang [10] MSAT error model, $P(\text{dropout})=1-P(\text{no dropout) }=2\rho_{1}\text{/}(1+\rho_{1})$, where *ρ*_1_ is the per-allele dropout rate. Dropout events for heterozygotes were modelled as binomial trials with the ‘success' probability equal to 2*ρ*_1_/(1 + *ρ*_1_). Subsequent to opportunity for dropouts, false allele events were modelled as binomial trials for each allele copy at a locus independently following the per-allele mistype rate, *ρ*_2_. Observed SNP genotypes were modelled following the generic typing error model outlined above where error events are treated as binomial trials for each allele copy at a locus separately following a per-allele rate, *γ* (electronic supplementary material, S1).

### 2.3. Match calling performance testing

Errors in clustering genetic mark–recapture samples into recaptures or unique specimens derive from errors in pairwise match calling. Thus, performance testing focused on the match calling model. For base case performance testing of the match calling model, input parameters for error rates and allele frequencies were equivalent to those from which data were simulated. We also examined a smaller suite of challenge trials to test the robustness of the match calling model to misspecifications in genotyping error rates and allele frequencies. Finally, we examined scenarios to test whether inclusion of poor-quality loci, i.e. with high genotyping error rates, would degrade match calling performance (table 1).

For each challenge trial, we simulated 10 000 comparisons for SNP data and 1000 comparisons for MSAT data (higher computation cost) of a pair of multilocus genotypes for data generated under each of three relationship states: SI, U and FS. Thus, performance was measured as the rate at which a given pair was correctly called a match (or non-match). Because genetic mark–recapture applications differ widely, we chose to assess match call error rates at the level of pairwise comparisons as this allows for generalization of performance outcomes to any sample matching scenario. For example, some studies may find a full-sibling false recapture error rate of 5%, where 1 in 20 times a pair of samples from two full siblings will be erroneously called a match, acceptable if the probability of actually sampling a pair of full siblings within the study population is extremely low. Owing to the high computation time required to implement the error-tolerant likelihood-based match calling model, we only considered SI, FS and *U* relationship states during simulation testing (i.e. excluding PO relationship state simulations).

### 2.4. Case studies

We assessed the error-tolerant likelihood-based matching approach and associated clustering algorithm to identify recaptures for both an SNP and MSAT case study. We first removed repeated captures of individuals within each sampling occasion using the stage-one sample clustering algorithm and then subsequently assessed recaptures across sampling occasions with the stage-two clustering algorithm. A total of four relationship states were assessed during clustering: R∈{SI, U, FS, PO} (electronic supplementary material, S2 and S3).

SNP case study data utilize pilot sampling for a genetic mark–recapture study implemented by the US Fish and Wildlife Service to assess the status of the Pacific walrus. Tissue samples were collected using dart-based biopsies, representing high-quality and high-quantity DNA samples. Data are available for 64 biallelic SNP markers, with pilot study samples from 2013 (initial ‘tag release’ sampling occasion) and 2014 (recapture sampling occasion). SNP genotyping was conducted by the US Fish and Wildlife Service Conservation Genetics Laboratory. Samples were run on four sets of TaqMan® OpenArray® Genotyping Plates, format 16, using the QuantStudio™ 12 K Flex Real-Time PCR System with the OpenArray® Block utilizing the Accufill™ System. One putative SNP failed, exhibiting a single allele, and was purged during subsequent analysis; thus sample matching was implemented with a maximum of 63 SNPs (electronic supplementary material, table S4.1). Approximately 100 individual samples were replicate genotyped three times (total replication numbers varied due to random PCR failures) and compared against per-sample consensus genotype calls to calculate per-locus generic genotyping error rates. Locus-level genotyping error-rate estimates varied across loci, some exhibiting zero errors. We implemented a 1% minimum locus-level genotyping error rate threshold during likelihood-based match calling and sample clustering, setting error rates to the empirically estimated values for loci with more than 1% error rates.

MSAT case study data are from two sampling occasions (henceforth referred to as occasions A and B) from a 2014 fisher genetic mark–recapture study in New York, USA [17]. Tissue was collected from follicles of hair samples collected from barbed wire snares, representing relatively lower quality and quantity DNA samples. Data are available for nine MSAT loci with a range of allelic dropout and false allele error rates (electronic supplementary material, table S4.2). Molecular details for MSAT data collection are described in electronic supplementary material, appendix S2 of Linden *et al*. [17]. Briefly, fluorescently labelled MSAT amplicons were analysed on an ABI 3730xl genetic analyser (Applied Biosystems) in the Cornell Institute of Biotechnology. Automated calling of genotypes was done with Genemapper 4.0 (Applied Biosystems) followed by manual checking of call accuracy. Locus-specific genotyping error rates were calculated using three replicate genotypes from each study sample (without regard to genotype quality; see [18]), estimating allelic dropout and false allele rates as per [19]. Samples with more than three loci with missing genotype calls were dropped from the sample match calling and clustering analysis, and a minimum locus-level genotyping error rate threshold of 0.5% for false allele events was imposed in specifying the match calling model.

## 3. Results

### 3.1. Simulation scenarios: base case results

**Figure 1.**
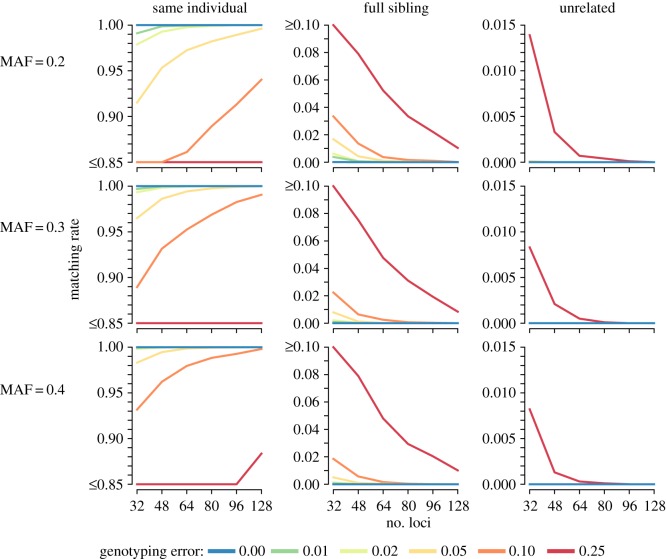
Simulation results of the error-tolerant likelihood-based match calling model for biallelic (SNP) markers. Plots are organized by minor allele frequency (MAF) along rows and true relationship state along columns. Results are the proportion of comparisons of 10 000 pairs of simulated samples from a given relationship state, minor allele frequency, number of loci and per-locus genotyping error rate that are called a match. Pairs of genotypes simulated from the ‘same individual’ relationship represent recapture events, for which match call rates of 1.00 are perfectly accurate and less than 1.00 indicate that missed recapture events occur; pairs of genotypes simulated from the ‘full sibling’ or ‘unrelated’ relationship states represent samples from different individuals, for which match call rates of 0.00 are perfectly accurate and more than 0.00 indicate that false recapture events occur. Note that *y*-axis values differ across columns.

The accuracy of SNP match calls to infer recaptures (*R* = SI; perfect accuracy indicated by match rate = 1.0) and non-match calls to identify unique specimens (R∈{U, FS}; perfect accuracy indicated by match rate = 0.0) improved with increasing numbers of loci (figure 1; electronic supplementary material, table S5.1). Accuracy increased with increasing minor allele frequencies; however, these improvements were marginal when compared with gains from increasing the number of loci. Non-match call accuracy was consistently strong when considering samples from unrelated individuals (figure 1, right column), where a 48-SNP panel produced perfect (non-match) call accuracy up to a very high 10% per-locus SNP genotyping error rate ([20,21]; electronic supplementary material, table S5.1). Non-match call accuracy for pairs simulated from full siblings was lower; however, false match calls for pairs of samples from full siblings occurred at less than a 1% rate for 64 SNP panels with up to a high 10% per-locus genotyping error rate. Finally, match call accuracy for recaptures (i.e. pairs of samples with *R* = SI) was more sensitive to genotyping error rates than was non-match call accuracy (i.e. pairs of samples with R∈{U, FS}). At the lower simulated per-locus error rates—of the order of 2% or less—panels of 48 SNPs or larger produced greater than 99.5% accuracy. At very high genotyping error rates in excess of 10% per locus, larger panels of the order of 128 SNPs (or greater) may be necessary to achieve match call accuracy of 95% or better (figure 1, left column).

**Figure 2.**
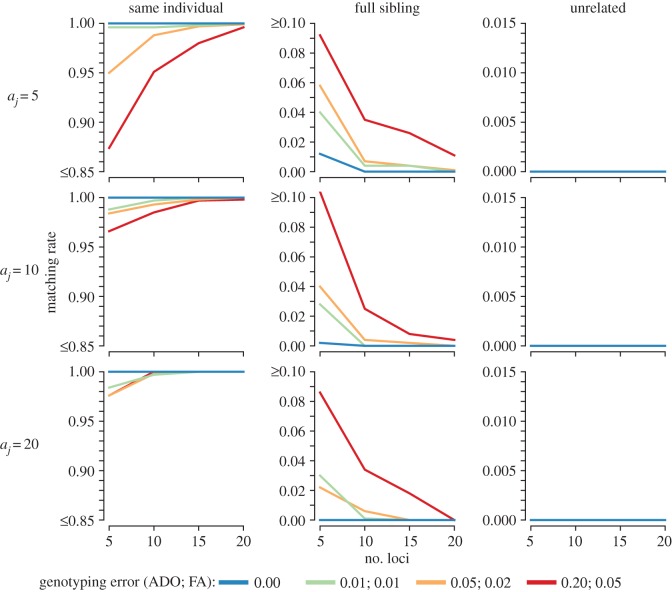
Simulation results of the error-tolerant likelihood-based match calling model for multiallelic (MSAT) markers. Plots are organized by the number of (equal frequency) alleles per locus, *a_j_*, along rows, and true relationship state along columns. Results are the proportion of comparisons of 1000 pairs of simulated samples from a given relationship state, number of loci, number of alleles and per-locus genotyping error rate that are called a match. Pairs of genotypes simulated from the ‘same individual’ relationship represent recapture events, for which match call rates of 1.00 are perfectly accurate and less than 1.00 indicate that missed recapture events occur; pairs of genotypes simulated from the ‘full sibling’ or ‘unrelated’ relationship states represent samples from different individuals, for which match call rates of 0.00 are perfectly accurate and more than 0.00 indicate that false recapture events occur. Note that *y*-axis values differ across columns.

MSAT markers exhibited high match call accuracy with moderately sized marker panels, although attaining very high non-match call accuracy for full-sibling pairs required somewhat larger panels of the order of 15 loci or greater (figure 2, middle column; electronic supplementary material, table S5.2). Similarly to simulations with biallelic SNP markers, MSAT markers demonstrated high non-match call accuracy for pairs of samples from unrelated individuals across the suite of marker panel and error rates simulated here (figure 2, right column). Increasing the number of equal-frequency alleles per locus improved non-match R∈{U, FS} and match call accuracy (*R* = SI); however, inclusion of additional loci had a more marked effect on accuracy. Finally, match call accuracy for pairs of samples from the same individual was somewhat less sensitive to genotyping error than for SNP panels, where MSAT match call accuracy for recaptures under 20% allelic dropout and 5% false allele per-locus error rates was greater than 95% for a small MSAT panel of five loci each with 10 equal-frequency alleles (match call rate = 0.966; figure 2, left column; electronic supplementary material, table S5.2).

### 3.2. Simulation scenarios: sensitivity analyses

Match call accuracy for both SNP and MSAT markers was robust to substantial deviations in allele frequency estimates from true latent frequencies, with both under- or overestimation of allele frequencies producing comparable and minor deviations from base case matching rates (tables 2 and 3). Furthermore, non-match calls when faced with samples from unrelated individuals remained error free across sensitivity tests. Sample matching was robust to inclusion of high-error rate loci, where match and non-match call accuracy actually improved marginally with the addition of poor-quality loci (per-locus generic typing error rate of 25% for SNP markers; 20% allelic dropout and 5% false allele rates for MSAT markers; tables 2 and 3).

**Table 2.** Match calling sensitivity analysis for SNPs.

	match call rate (difference from exact)^b^		
scenario^a^	*R* = FS	*R* = SI	*R* = U
***p*** overestimated, 48 loci, 1% latent genotyping error	*0.0000* (*−0.0001*)	0.9999 (0.0001)	**0.0000 (0.0000)**
***p*** overestimated, 64 loci, 1% latent genotyping error	**0.0000 (0.0000)**	**1.0000 (0.0000)**	**0.0000 (0.0000)**
***p*** overestimated, 48 loci, 10% latent genotyping error	0.0109 (0.0045)	0.9463 (0.0147)	**0.0000 (0.0000)**
***p*** overestimated, 64 loci, 10% latent genotyping error	0.0036 (0.0011)	0.9626 (0.0101)	**0.0000 (0.0000)**
***p*** underestimated, 48 loci, 1% latent genotyping error	*0.0000* (*−0.0001*)	*0.9996* (*−0.0002*)	**0.0000 (0.0000)**
***p*** underestimated, 64 loci, 1% latent genotyping error	**0.0000 (0.0000)**	**1.0000 (0.0000)**	**0.0000 (0.0000)**
***p*** underestimated, 48 loci, 10% latent genotyping error	0.0132 (0.0068)	0.9536 (0.022)	**0.0000 (0.0000)**
***p*** underestimated, 64 loci, 10% latent genotyping error	0.0056 (0.0031)	0.9668 (0.0143)	**0.0000 (0.0000)**
*γ* overestimated (latent: 1%, specified: 1.5%), 48 loci	**0.0001 (0.0000)**	1.0000 (0.0002)	**0.0000 (0.0000)**
*γ* overestimated (latent: 1%, specified: 1.5%), 64 loci	**0.0000 (0.0000)**	**1.0000 (0.0000)**	**0.0000 (0.0000)**
*γ* overestimated (latent: 10%, specified: 15%), 48 loci	0.0470 (0.0406)	0.9896 (0.0580)	**0.0000 (0.0000)**
*γ* overestimated (latent: 10%, specified: 15%), 64 loci	0.0262 (0.0237)	0.9956 (0.0431)	**0.0000 (0.0000)**
*γ* underestimated (latent: 1%, specified: 0.5%), 48 loci	*0.0000* (*−0.0001*)	*0.9978* (*−0.0020*)	**0.0000 (0.0000)**
*γ* underestimated (latent: 1%, specified: 0.5%), 64 loci	**0.0000 (0.0000)**	*0.9995* (*−0.0005*)	**0.0000 (0.0000)**
*γ* underestimated (latent: 10%, specified: 5%), 48 loci	*0.0002* (*−0.0062*)	*0.6515* (*−0.2801*)	**0.0000 (0.0000)**
*γ* underestimated (latent: 10%, specified: 5%), 64 loci	*0.0000 (−0.0025)*	*0.6622 (−0.2903)*	**0.0000 (0.0000)**
high-error loci included, 48 with 1% error and 16 with 10% error^c^	**0.0001 (0.0000)**	1.0000 (0.0002)	**0.0000 (0.0000)**
high-error loci included, 48 with 10% error and 16 with 25% error^c^	*0.0042* (*−0.0022*)	0.9319 (0.0003)	**0.0000 (0.0000)**

^a^See table 1 for additional scenario details. ***p* **= vector of allele frequencies; *γ* = per-locus genotyping error rate.

^b^Match rate call discrepancies are calculated as (scenario specific match rate – match rate from exactly specified input parameters); zero differences are bolded, negative differences are italicized. See table 1 for additional scenario details.

^c^Results are compared against a 48 SNP panel with 1% genotyping error.

**Table 3.** Match calling sensitivity analysis for MSATs.

	match call rate (difference from exact)^b^		
scenario^a^	*R* = FS	*R* = SI	*R* = U
***p*** specified incorrectly, 10 loci, ADO = 0.05, FA = 0.02	0.006 (0.002)	0.994 (0.001)	**0.000 (0.000)**
***p*** specified incorrectly, 15 loci, ADO = 0.05, FA = 0.02	0.003 (0.001)	0.999 (0.001)	**0.000 (0.000)**
***p*** specified incorrectly, 10 loci, ADO = 0.20, FA = 0.05	0.044 (0.019)	0.99 (0.005)	**0.000 (0.000)**
***p*** specified incorrectly, 15 loci, ADO = 0.20, FA = 0.05	0.011 (0.003)	0.997 (0.000)	**0.000 (0.000)**
error rates overestimated, 10 loci, latent ADO = 0.05, FA = 0.02	0.010 (0.006)	0.999 (0.006)	**0.000 (0.000)**
error rates overestimated, 15 loci, latent ADO = 0.05, FA = 0.02	*0.000* (*−0.002*)	1.000 (0.002)	**0.000 (0.000)**
error rates overestimated, 10 loci, latent ADO = 0.20, FA = 0.05	0.090 (0.065)	0.998 (0.013)	**0.000 (0.000)**
error rates overestimated, 15 loci, latent ADO = 0.20, FA = 0.05	0.057 (0.049)	1.000 (0.003)	**0.000 (0.000)**
error rates underestimated, 10 loci, latent ADO = 0.05, FA = 0.02	*0.000* (*−0.004*)	*0.977* (*−0.016*)	**0.000 (0.000)**
error rates underestimated, 15 loci, latent ADO = 0.05, FA = 0.02	*0.000 (−0.002)*	*0.994 (−0.004)*	**0.000 (0.000)**
error rates underestimated, 10 loci, latent ADO = 0.20, FA = 0.05	*0.006* (*−0.019*)	*0.912* (*−0.073*)	**0.000 (0.000)**
error rates underestimated, 15 loci, latent ADO = 0.20, FA = 0.05	*0.001* (*−0.007*)	*0.962* (*−0.035*)	**0.000 (0.000)**
high-error loci included, 10 loci with ADO = 0.05 and FA = 0.02, 5 loci with ADO = 0.20 and FA = 0.05^c^	*0.001* (*−0.003*)	0.999 (0.006)	**0.000 (0.000)**

^a^All simulations specify loci with 10 alleles. See table 1 for additional scenario details. ***p* **= vector of allele frequencies.

^b^Match rate call discrepancies are calculated as (scenario specific match rate – match rate from exactly specified input parameters); zero differences are bolded, negative differences are italicized.

^c^Results are compared against a 10 loci panel with ADO = 0.05 and FA = 0.02.

The greatest sensitivities of the error-tolerant likelihood-based match calling model arose when genotyping error rates were systematically underestimated, particularly for biallelic SNPs. The rate of correct match calls when faced with recapture samples for SNPs was biased strongly low when true genotyping error rates were high and specified error rates were low (table 2). The degree of impact from genotyping error rate misspecification was attenuated with larger SNP marker panels; however, downward bias in the match call rate for recapture samples persisted for 64 loci with 30% minor allele frequency when error rates were specified low (i.e. latent locus-level error rate = 10%; specified = 5%). MSAT markers also showed greatest sensitivity in match call accuracy in cases where genotyping error rates were specified lower than latent levels; however, the degree of impact on matching performance for recaptures was considerably less severe than for SNPs when latent error rates were high and specified rates were underestimated by 50% (table 3). Both marker types were robust to overestimation of genotyping error rates in terms of match calls for recapture pairs and non-match calls for distinct individuals. Together, these results suggest that conservative treatment of genotyping error rate specification may be warranted in order to avoid missed recaptures associated with underestimated genotyping error rates.

### 3.3. Case studies

Locus-level genotyping error rates for the Pacific walrus SNP case study data were in the range of 0.0–5.1% with all but eight of the 63 loci exhibiting error rates less than 1.0%, and 29 of 63 loci exhibiting 0 errors in replicate sampling (electronic supplementary material, table S4.1). Minor allele frequencies ranged from 0.493 to 0.124 (electronic supplementary material, table S4.1). The total panel (63 SNPs) probability of identity was 2.0 × 10^−26^ for unrelated individuals and 3.3 × 10^−14^ for full siblings. Repeated capture numbers within sampling occasions as assessed by the stage-one sample-clustering algorithm varied by a small number of individuals when sets of 32 (or 31) loci were used to make match calls, but stabilized when 47 or 63 SNP panels were utilized (table 4). All combinations of SNP panels ranging from 31 to 63 total loci identified the same set of eight recaptures across sampling occasions. The total number of loci in common with positive genotype calls (i.e. PCR amplification was successful and produced an unambiguous genotype call) for recapture samples varied in the range of 27–32 for 32-SNP panels, 43–47 for 47-SNP panels and 58–63 for 63-SNP panels; three of the recapture pairs had a single discrepant locus, whereas all others matched on all common positive loci (table 4). Likelihood ratios were large for recaptures and ranged from $\Lambda =1.12\;\times 10^{12}$ to $\Lambda =7.51\;\times 10^{13}$.

**Table 4.** Sample clustering results for Pacific walrus SNP multilocus genotype data^a^.

no. loci	within occasion recaptures 2013	within occasion recaptures 2014	between occasion recaptures^b^	range in common positive PCR loci for matches	maximum number of discrepancies observed in matches
31 (sets 1 and 2)	231	340	8	27–31	1
32 (sets 1 and 3)	231	343	8	27–32	1
31 (sets 2 and 3)	237	347	8	27–31	1
47 (sets 1–3)	231	336	8	43–47	1
63 (sets 1–4)	231	336	8	58–63	1

^a^SNPs are grouped into sets of 16; one locus in set 2 manifested a single allele and was purged from the analysis. A locus-level genotyping error floor of 1% was imposed for all loci during the likelihood-based error-tolerant matching protocol (see electronic supplementary material, table S4.1 for empirical rates).

^b^All SNP set combinations identified the same individuals as recaptures across sampling occasions.

Locus-level genotyping error rates for the fisher MSAT case study were in the range of 8.0–17.0% for allelic dropout events and 0.6–2.0% for false allele mistyping events (electronic supplementary material, table S4.2). Loci had five to eight alleles, generally with two to three common alleles and several rare, with a total marker panel probability of identity of 9.6 × 10^−8^ for unrelated individuals and 1.0 × 10^−3^ for full siblings. Repeated captures within sampling occasions as assessed by the stage-one sample clustering algorithm occurred at a similar rate for both periods, generating 18 repeat captures during occasion A (18/95 = 18.9% repeat capture rate) and 23 during occasion B (23/105 = 20.9% repeat capture rate). A total of 18 recaptures were identified across the two sampling occasions. Because the marker panel contained relatively low discriminating information and non-negligible genotyping error rates, we reran the clustering algorithm across the sampling occasions (‘stage-two’ clustering, see Material and methods) two additional times after permuting the order of samples and found the same recapture events identified in all cases. Similarly to the walrus case study, not all recapture pairs had a full complement of loci with positive genotype calls, with the number of positive loci in common ranging from six to nine for fisher recaptures. Nine recapture pairs had one or more discrepant genotype calls. Likelihood ratios for recaptures were much lower for fisher MSAT data than for walrus SNP data, ranging from Λ=6.1 to Λ=7498.7.

## 4. Discussion

The error-tolerant likelihood-based match calling model performed well in simulation trials when specified allele frequencies and genotyping error rates matched those from data generation. In empirical applications, allele frequencies and genotyping error rates are estimated from samples of the population of interest and thus contain estimation errors. Sensitivity analysis simulations demonstrate robustness of the error-tolerant likelihood-based match calling model to errors in specifying allele frequencies. Match calling performance was also found to be robust to small genotyping error rate misspecifications; however, large genotyping error rate misspecifications were indicated to be more problematic. Thus, it may be advantageous to conduct ample replicate genotyping to establish accurate genotyping error rates, as well as to remain conservative in specifying error rates. In particular, zero genotyping error rate estimates, which may occur when true genotyping error rates are low and replication for the purposes of estimating error rates is also low, provide strong information to the match calling model which may not reflect reality.

A primary advantage of the error-tolerant likelihood-based matching approach is that a strength of evidence approach is used to assess matches in the face of possible genotyping errors. For example, a number of recaptures in both case studies had at least one discrepant locus in a match call, illustrating how genotyping error can persist even after conventional measures are taken to minimize it. An important feature of this approach is that matching performance always remained stable or improved as the genetic marker panel size increased, even if additional loci were error-prone. Many studies filter out error-prone samples in order to avoid passing genotyping errors through to sample matching. While quality control and good laboratory practices are essential for any successful genetic mark--recapture study (e.g. [5]), error-tolerant matching protocols such as tested here may make it possible to apply less stringent filters in the laboratory and include more of the collected samples in the final sample- matching analysis.

Based upon simulation results, high accuracy match and non-match calls can be reached with reasonably sized marker panels (e.g. 64+ SNPs, 10–15 MSATs), ultimately leading to accurate grouping of samples into recapture sets during sample clustering. However, even with low matching error rates, analysts may wish to remain conservative in the choice of panel size because the clustering algorithm requires that all pairwise combinations between samples be assessed in making match calls. The total number of pairwise comparisons is the appropriate number of match call comparisons for a given relationship state to consider when targeting match call error rates. For example, under the clustering algorithm proposed here, the number of pairwise comparisons made in assessing recaptures across two occasions each with 500 samples is 500^2^ = 250 000, and thus one would need to achieve a generic match call error rate below 1/250 000 = 4.0 × 10^−6^ to avoid making at least one expected match call error. Fortunately, simulations suggest that the error-tolerant likelihood-based matching protocol yielded near-perfect accuracy in making non-match calls for unrelated individuals—a relationship state that probably characterizes most pairwise comparisons from a wide range of populations to be assessed with genetic mark–recapture—at reasonably sized marker panels. To illustrate this point, consider the artificial case of a single sampling occasion sample size of 500 individuals made up of 250 pairs of full siblings. In total, there are $\left(\begin{array}{c} 500\\ 2 \end{array}\right)=124,750$ different pairwise comparisons of samples to be made in a single iteration of identifying within sampling occasion recaptures, where the notation $\left(\begin{array}{c} x\\ y \end{array}\right)$ indicates *x* choose *y*. Of these pairwise comparisons, $250\times \left(\begin{array}{c} 2\\ 2 \end{array}\right)=250$ are pairs of full siblings, requiring a target full-sibling pairwise comparison non-match call accuracy rate of 1/250 = 0.004 to avoid making at least one expected false full-sibling match call. Furthermore, particularly with non-invasive genetic sampling such as hair or scat collection, larger marker panels may be warranted given that negative PCR outcomes will often reduce the number of loci with common positive genotype calls when comparing a pair of multilocus genotypes. For example, even with high-quality tissue biopsy samples for the walrus case study, most recapture samples had fewer loci with positive genotype calls than the full complement of loci available (table 4).

Genetic mark–recapture provides substantial advantages in the field by allowing cryptic or hard-to-handle taxa to be sampled; however, this benefit comes at the cost of having to infer recapture events from genotypes with genotyping errors as opposed to directly observing recaptures in traditional physical- or image-based recapture studies. To facilitate design of genetic mark–recapture studies and to assess whether a given genetic marker panel and field design are sufficient to produce acceptable mark–recapture modelling performance, we suggested a protocol to simulate mark–recapture inferences based on identification of recaptures with error-prone genotypes. Using parameters drawn from a specific marker panel, analysts can simulate genotypes under genotyping error and assess match calling and sample clustering performance. Simulation results indicate the frequency of false recaptures and missed recapture outcomes as a function of genotyping error rate, sample size and recapture rate anticipated for a given genotyping and field sampling design (e.g. electronic supplementary material, S2 and S3). Alternatively, less involved though potentially less precise, match and non-match call accuracy rates from simulation results presented in figures 1 and 2 (also see electronic supplementary material, S5) can be multiplied by the number of pairwise comparisons needed to complete sample clustering for sample sizes and recapture rates anticipated with a given field design in order to predict the incidence of false recaptures and missed recaptures. Subsequently, the expected false recapture and missed recapture rates can be incorporated into simulations of mark–recapture estimation to assess the impact of the anticipated recapture identification error rates on key parameters of interest, such as estimated abundance (e.g. [4,22,23]). Should biases in mark–recapture parameter estimates introduced by recapture errors be found to be unacceptable, analysts could repeat the proposed simulation process by exploring different options to improve recapture identification from genotypes (e.g. replicate genotyping to reduce genotyping error or inclusion of additional loci).

A pragmatic challenge in implementing this match calling and sample clustering protocol is that computational cost can be high when loci have high allelic richness. For example, evaluation of the likelihood of a given relationship state for a pair of diploid genotypes requires summation of calculations over all possible unordered pairs of latent genotypes, which for a single locus is equal to ${\left[\left(\begin{array}{c} a_{j}+1\\ 2 \end{array}\right)\right]}^{2}$. With 20 loci each with 20 alleles, this equates to 882 000 sums for evaluation of a single relationship state hypothesis for one multilocus genotype pair comparison. Based solely on a criterion of computation cost, biallelic SNP markers have a considerable advantage over MSAT markers with higher allelic richness, in that a total of only nine sums per biallelic locus need be made in comparing a pair of multilocus genotypes. Regardless, to speed up the clustering algorithm to group samples into putative recapture sets, it may be helpful to include a bypass point to avoid comparing multilocus genotypes which have a high number of discrepant genotype calls across loci and thus would have very low probability of having come from the same individual (e.g. [12,15,24]). We caution, however, that in using such an approach, a conservative loci-mismatch threshold be utilized to avoid introducing missed recapture errors by failing to identify recapture pairs which by chance exhibited a large number of genotyping errors (cf. [12]).

As genomic techniques advance, the development and genotyping costs for large marker panels will continue to decrease (e.g. [25,26]). MSAT marker panels performed well in simulation trials; however, SNPs are computationally more efficient during the match calling and sample clustering protocol. We anticipate increasingly large panels of SNPs or similar markers will be available, enabling arbitrarily high match call accuracy and subsequent recapture history reconstruction using error-tolerant likelihood-based match calling and sample clustering algorithms, although computational efficiency will present challenges as genotyping data increases in volume.

## Supplementary Material

Supplementary Materials are combined into a single .pdf document, with the following contents: Supplement 1: Detail of the error-tolerant likelihood-based match calling and sample clustering approach Supplement 2: R script to implement the error-tolerant likelihood-based match calling model and sample clustering algorithms: MSATs Supplement 3: R script to implement the error-tolerant likelihood-based match calling model and sample clustering algorithms : SNPs Supplement 4: Case study genetic marker characteristics Supplement 5: Detailed base case simulation results

## Data accessibility

R code to support simulation and case study analyses, and genetic marker information to support case study results in this article have been uploaded as part of the electronic supplementary material. Primary walrus genetic information are archived by and publicly available from the U.S. Fish and Wildlife Service Marine Mammals Management division, Alaska, www.fws.gov/alaska. Primary fisher genetic data are publicly available from Linden *et al.*, forthcoming, doi: http://dx.doi.org/10.1101/066662.
