# Supplementary material for: Accurate recapture identification for genetic mark–recapture studies with error-tolerant likelihood-based match calling and sample clustering
Source: R Soc Open Sci. 2016 Dec 21;3(12):160457. doi: 10.1098/rsos.160457 (PMC5210676; doi:10.1098/rsos.160457)
Supplement: Supplementary Materials are combined into a single .pdf document, with the following contents: Supplement 1: Detail of the error-tolerant likelihood-based match calling and sample clustering approach Supplement 2: R script to implement the error-tolerant likelihood-based match calling model and samp [file rsos160457supp1.pdf]

Accurate recapture identification for genetic mark-recapture studies with error-tolerant likelihood-based match calling and sample clustering.

Supplementary Materials

Suresh A. Sethi*, Daniel Linden, John Wenburg, Cara Lewis, Patrick Lemons, Angela Fuller, Matthew P. Hare

*e.: suresh.sethi@cornell.edu

Contents:

Supplement 1: Detail of the error-tolerant likelihood-based match calling and sample clustering approach
Supplement 2: R script to implement the error-tolerant likelihood-based match calling model and sample clustering algorithms: MSATs
Supplement 3: R script to implement the error-tolerant likelihood-based match calling model and sample clustering algorithms : SNPs
Supplement 4: Case study genetic marker characteristics
Supplement 5: Detailed base case simulation results

Supplement 1: Detail of the error-tolerant likelihood-based match calling and sample clustering approach

Following methods developed by Wang (2004, 2006, 2016) and Kalinowski et al. (2006), the error-tolerant likelihood-based match calling and sample clustering approach contains four components: 1) a model for observed genotypes which accommodates genotyping error, 2) a model to describe the probability of obtaining a pair of true "latent" genotypes, 3) a likelihood model to evaluate the evidence to call a match between a pair of observed genotypes, and 4) a sample clustering algorithm to group genotypes into same-individual sets, i.e. recapture events. We utilized the genotyping error model for microsattelite (MSAT) genotypes from Wang (2004,2006,2016) and propose a separate genotyping error model for single nucleotide polymorphism (SNP) genotypes. Statements to inform the joint probability of observing a pair of latent genotypes needed to implement the likelihood-based match calling model proposed analogously by Kalinowski et al. (2006) and Wang (2016) were taken from Weir et al. (2006). The formulation of the likelihood for an observed pair of genotypes coming from a given relationship state (e.g. "same individual", indicating a recapture event) follows that analogously proposed by Kalinowski et al. (2006) and Wang (2016), however, we utilize likelihood ratios to make match calls, as presented in Kalinowski et al. (2006). Finally, we propose a novel two-stage sample clustering approach. Table S1.1 provides notation definitions.

The assumptions of the error-tolerant likelihood-based match calling model (LM) as implemented here are as follows:

A1. The sample population is non-inbred and mating is random.
A2. Genotypes are diploid with codominant alleles.
A3. Loci are independent (non-linked).
A4. Estimates of allele frequencies are unbiased.
A5. The genotyping error process proposed during probability calculations matches that from the genotyping error data generating process for sample genotypes
A6. Estimates of genotyping error rates are unbiased.

Assumptions A1-A2 are necessary for the specification of probabilities of experiencing a pair of multilocus genotypes given a relationship state and population allele frequencies. Statements for inbred populations are also available (e.g. Weir et al. 2006). With Assumption A3, joint probabilities of observed multilocus genotypes can be computed by multiplying across loci. Assumptions 4-6 are necessary to ensure the specified probability models are consistent with the multilocus genotype data generating process, although the LM approach demonstrates some robustness to deviations from these assumptions (see the Main Text).

Describing the four components to the LM and sample clustering approach in detail, first, a model for the probability of observing a sampled genotype at locus j for individual i, g_{ij}, given a proposed latent genotype, k_{ij}, $P(g_{ij}|k_{ij})$ is specified. In the present implementation, we specified a two-class genotyping error model for MSAT markers following Wang (2004, 2006, 2016) and comprising of an allelic dropout rate, $\rho_{j,1}$, and a false allele (mistyping) error rate, $\rho_{j,2}$, where error rates are *per allele*. Under Wang's genotyping error model, allelic dropout events occur before false allele events, and either allele in a diploid genotype is equally probable to dropout. Subsequent to allelic dropout, 0, 1, or 2 false allele (i.e. mistyping) events can occur. For a_j alleles at locus j, when a false allele mistype occurs, it is assumed that all alleles have equal probability of being called. For notational convenience (sensu Wang 2004, 2006, 2016), define $r_{j,1} = {\rho_{j,1}}/{(1+\rho_{j,1})}$ and $r_{j,2} = {\rho_{j,2}}/{(a_j - 1)}$. Note, the allelic dropout rate is relevant to latent heterozygotes only, such that empirical allelic dropout rates should be estimated based upon attempts to genotype heterozygotes; false allele events apply to both latent heterozygotes and homozygotes and thus false allele error rates should be estimated based upon the total number of genotypes attempted for error rate estimation (see Broquet and Petit 2004). Dropping for the moment individual and locus subscripts, let $g_{\{u,v\}}$ indicate an observed genotype with alleles u and v, and let $k_{\{w,x\}}$ indicate a latent genotype with alleles w and x. From Wang (e.g. 2016), combining allelic dropout and false allele events, then for latent heterozygote genotypes ($w \neq x$):

$$P(g_{\{u,v\}}|k_{\{w,x\}}) = \begin{cases} (1-\rho_2)^2 + r_2^2 - 2r_1(1-r_2-\rho_2)^2 \\ r_2(1-\rho_2) + r_1(1-\rho_2-r_2)^2 \\ r_2^2 \\ 2r_2^2 \\ r_2(1-\rho_2-r_2) \end{cases} \text{for } \begin{matrix} u=w, v=x \\ u=v=w, \text{or } u=v=x \\ u\neq w, u\neq x, v\neq w, v\neq x \text{ with } u=v \\ u\neq w, u\neq x, v\neq w, v\neq x \text{ with } u\neq v \\ \text{otherwise} \end{matrix} \text{ i.e. } \begin{matrix} \text{No errors} \\ \text{Apparent allelic dropout} \\ \text{2 mistypes, same allele} \\ \text{2 mistypes, different alleles} \\ \text{1 mistype} \end{matrix},$$

and for latent homozygous genotypes ($w = x$):

$$P(g_{\{u,v\}}|k_{\{w,x\}}) = \begin{cases} (1-\rho_2)^2 \\ 2r_2(1-\rho_2) \\ r_2^2 \\ 2r_2^2 \end{cases} \text{for } \begin{matrix} u=v=w=x \\ u=w, v\neq w \text{ or } v=w, u\neq w \\ u\neq w, v\neq w \text{ with } u=v \\ u\neq w, v\neq w \text{ with } u\neq v \end{matrix} \text{ i.e. } \begin{matrix} \text{No errors} \\ \text{1 mistype} \\ \text{2 mistypes, same allele} \\ \text{2 mistypes, different alleles} \end{matrix}.$$

Note, in some cases probability statements account for multiple pathways between latent genotypes and observed genotype outcomes; for example, what appears to be an allelic dropout event could occur because one allele from a latent heterozygote genotypes drops out during genotyping, or from a mistype event where the mis-called allele happens by chance to mirror the other allele copy (among other pathways).

For SNP markers, we assume loci are bi-allelic, whereby we implement a simple generic genotyping error model to reflect that a given allele is either called correctly as the latent allele or incorrectly as the opposite allele. Dropping locus and individual subscripts, let d_{gk} be the total number of alleles discrepant between observed genotype g and latent genotype k. The probability of observing g given a proposed k is,

$$P(g|k) = \gamma^{d_{gk}}(1-\gamma)^{2-d_{gk}},$$

where γ is the *per allele* genotyping error rate. Under this error model for biallelic SNPs, when an error occurs the latent allele is switched for the other existing allele at the locus. A total of 0 (e.g. $(g,k) = (AB, AB)$), 1 (e.g. $(g,k) = (AB, BB)$), or 2 (e.g. $(g,k) = (AA, BB)$) miss-called alleles can occur at a locus, i.e. $d_{gk} \in \{0,1,2\}$.

Second, a model is proposed for the probability of observing an unordered pair of latent diploid genotypes, $P(k_1, k_2|R)$, given a proposed relationship state, R (e.g. $R \in \{SI, U, FS, PO\}$; SI = a pair of samples from the same individual, U = samples from unrelated individuals, FS = samples from a pair of full siblings, and PO = samples from a parent offspring pair; other relationships could also be assessed if desired), and set of allele frequencies $\boldsymbol{p}_j = \{p_{j,1}, \dots, p_{j,a_j}\}$. Let u, v, w, and x denote four alleles. Dropping subscripts for individuals and loci and utilizing equations provided in Weir et al. (2006) for non-inbred populations, joint probabilities of experiencing a pair of latent genotypes in the population are:

$$P(k_1,k_2|R) = \begin{cases} y_2 p_u^2 + y_1 p_u^3 + y_0 p_u^4 & uu,uu & \text{2 hom., 2 shared alleles} \\ y_0 p_u^2 p_v^2 & uu,vv & \text{2 hom., 0 shared alleles} \\ y_1 p_u^2 p_v + 2 y_0 p_u^3 p_v & uu,uv & \text{1 hom., 1 het., 1 shared allele} \\ 2 y_0 p_u^2 p_w p_x & \text{when } uu,wx & \text{i.e. 1 hom., 1 het., 0 shared allele} \\ 2 y_2 p_u p_v + y_1 p_u p_v (p_u + p_v) + 4 y_0 p_u^2 p_v^2 & uv,uv & \text{2 het., 2 shared alleles} \\ y_1 p_u p_v p_x + 4 y_0 p_u^2 p_v p_x & uv,ux & \text{2 het., 1 shared alleles} \\ 4 y_0 p_u p_v p_w p_x & uv,wx & \text{2 het., 0 shared alleles} \end{cases}$$

where probabilities of 0, 1, or 2 alleles identical by descent, y_0, y_1, and y_2, respectively, given a relationship state are defined as:

$$y_2 = \begin{cases} 1 \\ 0 \\ 0.25 \\ 0 \end{cases} \text{for } R = \begin{matrix} SI \\ U \\ FS \\ PO \end{matrix}, \; y_1 = \begin{cases} 0 \\ 0 \\ 0.5 \\ 1 \end{cases} \text{for } R = \begin{matrix} SI \\ U \\ FS \\ PO \end{matrix}, \text{ and } y_0 = \begin{cases} 0 \\ 1 \\ 0.25 \\ 0 \end{cases} \text{for } R = \begin{matrix} SI \\ U \\ FS \\ PO \end{matrix}.$$

Note, depending on the number of alleles at a locus, not all latent genotype pair outcomes are possible. For example, for biallelic SNPs, there are only 4 possible outcomes for (unordered) pairs of latent diploid genotypes: $\{uu, uu\}$, $\{uu, vv\}$, $\{uu, uv\}$, and $\{uv, uv\}$.

Third, the likelihood of a hypothesized relationship state between a pair of sample multilocus genotypes, $R_{(1,2)}$, which incorporates information on population allele frequencies as well as genotyping error is calculated as:

$$L\left(R_{(1,2)}\right) = \prod_j \left[\sum_{k_{j,1}}^{A_j} \sum_{k_{j,2}}^{A_j} P\left(k_{j,1}, k_{j,2} | R_{(1,2)}\right) P\left(g_{j,1} | k_{j,1}\right) P\left(g_{j,2} | k_{j,2}\right)\right],$$

where indexing for summations indicate that sums are taken across the set of all possible latent diploid genotypes at locus j, A_j. The strength of evidence for the SI relationship state, i.e. a match call, is assessed by calculating the ratio of likelihood of a SI relationship to the maximum likelihood alternative relationship hypothesis:

$$\Lambda = L(R_{(1,2)} = SI) / \max\left\{L\left(R_{(1,2)} = U\right), L\left(R_{(1,2)} = FS\right), L(R_{(1,2)} = PO)\right\}.$$

If $\Lambda > 1.0$, then a match call is made, else the samples are inferred to have come from separate individuals. The framework developed by Kalinowski et al. (2006) allows for multiple captures to be grouped within a single sample cluster in assessing match calls, for instance as multilocus genotypes would reflect if a single individual is repeatedly sampled three or more times within a sampling occasion. In such cases the above likelihood model is expanded to consider pairwise comparisons of sets of multilocus genotypes, G_1 and G_2, as opposed to pairwise comparison of single multilocus genotypes. In this case, $L\left(R_{(G_1,G_2)}\right)$ is defined as:

$$L\left(R_{(G_1,G_2)}\right) = \prod_j \left[\sum_{k_{j,1}}^{A_j} \sum_{k_{j,2}}^{A_j} P\left(k_{j,1}, k_{j,2} | R_{(G_1,G_2)}\right) P\left(G_{j,1} | k_{j,1}\right) P\left(G_{j,2} | k_{j,2}\right)\right]$$

where $P\left(G_{j,1} | k_{j,1}\right) = \prod_{g_j \in G_{j,1}} P\left(g_{j,1} | k_{j,1}\right)$ indicates that probabilities of observed genotypes in a set given a latent genotype are the product across $P\left(g_{j,1} | k_{j,1}\right)$ for all genotypes in set G_1 at locus j. Subsequently, the likelihood ratio to evaluate the strength of evidence for the SI relationship state is calculated as above.

Finally, a sample clustering algorithm is implemented to group multilocus genotypes into singleton sets indicating unique individuals and sets of recaptures. We implement a two-stage sample clustering approach to identify recapture events with the error-tolerant likelihood-based match calling model for genetic mark-recapture studies. In stage one, a clustering algorithm is implemented to identify repeated captures within a single sampling occasion. Given a list of size n_1 genotype sets from a sampling occasion, $S_1 = \left(G_1, \ldots, G_{n_1}\right)$ ordered with an indexing sequence of $z = (1, \ldots, n_1)$:

Step 1: Define $S = S_1$ with each sample in S_1 as a singleton set.
Step 2: Compare the first genotype set in the list, G_1 against all other genotype sets in S, G_z for $z > 1$, and combine sets into G_1 as a match when $\Lambda > 1.0$.

Step 3: Compare the next genotype in sequence, e.g. G_2, against all other remaining sets in S, G_z for $z > 2$, combining sets as a match when $\Lambda > 1.0$, and repeat until the last genotype set in sequence is reached, generating an updated set of genotypes, $\tilde{S} = (\tilde{G}_1, \dots, \tilde{G}_{\tilde{n}_1})$, where $\tilde{n}_1 \leq n_1$.
Step 4: Repeat Steps 2-3 with $S = \tilde{S}$; if no set memberships change, stop, else repeat this step.

After completion of this algorithm, sets within $\tilde{S}$ with 2 or more genotypes indicate repeated captures within a sampling occasion and can be condensed into a single unique multilocus genotype (possibly reconstructing consensus genotypes from repeated captures of the same individual). Stage one clustering would be implemented for each sampling occasion in the mark-recapture study. Subsequently, a second stage algorithm is implemented to identify recaptures across sampling occasions' lists of unique individuals, $\tilde{S}_1 = (G_{1,1}, \dots, G_{1,\tilde{n}_1})$ and $\tilde{S}_2 = (G_{2,1}, \dots, G_{2,\tilde{n}_2})$:

Step 1: Compare the first genotype set in sequence in $\tilde{S}_1$ against all genotype sets in $\tilde{S}_2$ and combine sets as a match when $\Lambda > 1.0$. Sets from $\tilde{S}_2$which are combined into a given set in $\tilde{S}_1$ are removed from $\tilde{S}_2$.
Step 2: Compare the next genotype in sequence in $\tilde{S}_1$ against all remaining sets in $\tilde{S}_2$ combining sets as a match when $\Lambda > 1.0$ as in Step 1, and repeat until the last genotype set in sequence in $\tilde{S}_1$ is compared against all remaining sets in $\tilde{S}_2$ generating updated sets of genotypes, S_1^* and S_2^*.

After this second stage clustering, sets in S_1^* with two genotypes indicate recapture events; singleton sets in S_1^* and S_2^* represent unique individuals not recaptured across the pair of compared sampling occasions. Note, this version of the clustering algorithm assumes the starting sample sets $\tilde{S}_1$ and $\tilde{S}_2$ are made up solely of unique individuals. In this case, any given individual can only be recaptured once across a pair of compared sampling occasions' lists of unique genotypes and only a single iteration of the algorithm is necessary. Stage two clustering would be implemented for each pairwise comparison of sampling occasions within the mark recapture study and results ultimately translated to individual capture histories.

References:
Broquet T, Petit E. 2004 Quantifying genotyping errors in noninvasive population genetics. *Molecular Ecology* **13**, 3601-3608.
Kalinowski ST, Taper ML, Creel S. 2006 Using DNA from non-invasive samples to identify individuals and census populations: an evidential approach tolerant of genotpying errors. *Conservation Genetics* **7**, 319-329.
Wang J. 2004 Sibship reconstruction from genetic data with typing errors. *Genetics* **166**, 1963-1979.
Wang J. 2006 Informativeness of genetic markers for pairwise relationship and relatedness inference. *Theoretical Population Biology* **70**, 300-321.
Wang J. 2016 Individual identification from genetic marker data: developments and accuracy comparisons of methods. *Molecular Ecology Resources* **16**, 163-175.
Weir B, Anderson AD, Hepler AB. 2006 Genetic relatedness analysis: modern data and new challenges. *Nature Reviews Genetics* **7**, 771-780.

Table S1.1 Notation for the error tolerant likelihood-based clustering sample matching approach[a].

Notation	Definition
g_{ij}	An observed diploid genotype for individual i at locus j.
k_{ij}	A latent diploid genotype for individual i at locus j.
$\rho_{j,1}$	Per allele rate of allelic dropout errors (Class I error rate; Wang 2004, 2016) for the MSAT genotyping error model.
$\rho_{j,2}$	Per allele rate of false allele errors (Class II error rate; Wang 2004, 2016) at locus j for the MSAT genotyping error model.
$r_{j,1}$	Placeholder variable for the MSAT genotyping error model, equal to $\rho_{j,1} / (1+\rho_{j,1})$
a_j	Number of alleles at locus j.
$r_{j,2}$	Placeholder variable for the MSAT genotyping error model, equal to $\rho_{j,2} / (a_j - 1)$
d_{gk}	Total number of allele discrepancies at a locus between an observed and latent diploid genotype for the SNP genotyping error model.
γ	The per-allele mistyping rate for the SNP genotyping error model.
R	A hypothesized relationship state, $R \in \{SI, U, FS, PO\}$.
$\boldsymbol{p}$	A vector of a allele frequencies at a locus, $\{p_1, \ldots, p_a\}$.
y_0, y_1, and y_2	Probabilities of experiencing 0, 1, or 2 allele identical by descent at a diploid locus.
$R_{(1,2)}$	A relationship state hypothesis when comparing a pair of multilocus genotypes.
A_j	Set of all possible diploid latent genotypes at locus j.
Λ	Maximum likelihood ratio for the strength of evidence of $R_{(1,2)} = SI$, i.e. a "match" considering alternative hypotheses of $R_{(1,2)} = U$, $R_{(1,2)} = FS$, and $R_{(1,2)} = PO$.
G_1	A set of multilocus genotypes (possibly a singleton set).
$R_{(G_1,G_2)}$	A relationship state hypothesis when comparing a pair of sets of multilocus genotypes
$S, \tilde{S}, S^*$	Ordered lists of sets of multilocus genotypes; clustering notation. For example, $S_1 = (G_{1,1}, \ldots, G_{1,n_1})$.
n_1	Number of samples in set S_1

[a]Acronyms: MSAT = microsatellite, SNP = single nucleotide polymorphism, SI = same individual, U = unrelated, FS = full sibling, PO = parent offspring.

Supplement 2: R script to implement the error-tolerant likelihood-based match calling model and sample clustering algorithms: MSATs

```
# Example code to conduct likelihood-based error-tolerant sample matching and clustering for
# genetic mark recapture studies: multiallelic markers for diploid genotypes.

# Version 1.0-Nov09_2016

# Sethi SA, Linden D, Wenburg J, Lewis C, Lemons P, Fuller A, Hare M

# Overview: This script contains code to implement likelihood-based error-
# tolerant "MSAT" genotype matching, and subsequent stage 1 (within a sampling occasion)
# and stage 2 (between sampling occasions) clustering to identify recaptures.
# See Supplement 1 of Sethi et al. for explanation of methods and description
# of formulae.  Below, references are provided indicating source articles from which
# probability models and genotyping error models were derived.
# The intent of this script is to provide example code
# with which to implement likelihood-based error-tolerant genotype matching
# and sample clustering for genetic mark recapture data, however this code is
# not maintained by the authors.

# Contents:
# Section 1: User defined functions
# Section 2: Example data
# Section 3: Stage one clustering to identify repeated captures within a single sampling occasion
# Section 4: Stage two clustering to identify recaptures across sampling occasions
# Section 5: References

### Section 1: User defined functions (load these first) ############################################################

  #-----------------------------------------------------------------------------------------------------------#
  # User defined function to calculate the probability of observing a sample genotype at locus j
  # given a proposed true latent genotype, Pr(g_ij|k_ij).

    # Microsatellite (MSAT) two-class genotyping error model from Wang (2004, 2006, 2016)
      # P.error.msat() -- Calculates the probability of observing a sample diploid genotype at locus
      # j given a true latent genotype k for multiallelic (microsatellite) type markers.
      # Two classes of error: allelic dropout (e.g. observed AA from true Aa) and false alleles
      # (mistype, e.g. observed Ab from AA). Under Wang's MSAT error model, the following
      # assumptions are made:
        # a) same per-allele dropout rate and per-allele mistype rate for all alleles at j,
        # b) only one dropout possible per locus,
        # c) up to two mistype errors can occur per locus,
        # d) mistyping errors occur after possibility of dropouts.
      # Arguments: rho1 = per-allele allelic dropout rate at locus j; rho2 = per-ALLELE false
      # allele (mistype) rate at locus j; a = number of alleles at locus j; gj = observed diploid
      # genotype at j; kj = true latent genotype at j. Allelic dropout rates are relevant to true latent
      # heterozygotes; false allele rates are relevant to both latent heterozygotes and homozygotes,
      # e.g. see Broquet and Petit (2004).
      # Input genotypes: NOTE, input genotypes must be two-character strings (e.g. "ab","Bc") and if using
      # roman letters, this code is case sensitive.  Pairs of alleles are unordered, e.g. "aB" equivalent to "Ba"
      # Please code multiallelic loci with many alleles carefully. Coding alleles as lower and upper case
      # letters and single numeric digits 0-9, can accommodate loci with 26+26+10 = 62 alleles, and could be
      # expanded further if need be with other special characters read in as strings beyond that (e.g. "!" or "@").
        P.error.msat <- function(rho1,rho2,a,kj,gj){
          r1 <- rho1/(1+rho1)
          r2 <- rho2/(a-1)
```

```
      out.msat <- 1
      if(length(unique(strsplit(kj,"")[[1]]))==1){ # homozygous latent
        if(adist(kj,gj)==0){out.msat <- (1-rho2)^2} else # no error manifests
        if((2-sum(unique(strsplit(kj,"")[[1]]) %in% unique(strsplit(gj,"")[[1]])))==1 &
          adist(kj,gj)>=1){out.msat <- 2*r2*(1-rho2)} else # one mistype
        if((2-sum(unique(strsplit(kj,"")[[1]]) %in% unique(strsplit(gj,"")[[1]])))==2 &
          length(unique(strsplit(gj,"")[[1]]))==2){out.msat <- 2*r2^2} else # two mistypes, different allele manifests
        if((2-sum(unique(strsplit(kj,"")[[1]]) %in% unique(strsplit(gj,"")[[1]])))==2 &
          length(unique(strsplit(gj,"")[[1]]))==1){out.msat <- 1*r2^2} # two mistypes, same allele manifests
        } else # end homozygote latent genotype and proceed to heterozygote latent
      {
        if((2-sum(unique(strsplit(kj,"")[[1]]) %in% unique(strsplit(gj,"")[[1]])))==0){
          out.msat <- (1-rho2)^2 + r2^2 - 2*r1*(1-rho2-r2)^2 } else # no error manifests
        if((2-sum(unique(strsplit(kj,"")[[1]]) %in% unique(strsplit(gj,"")[[1]])))==1 &
          length(unique(strsplit(gj,"")[[1]]))==1){out.msat <- r2*(1-rho2) + r1*(1-rho2-r2)^2} else # allelic dropout
        if((2-sum(unique(strsplit(kj,"")[[1]]) %in% unique(strsplit(gj,"")[[1]])))==1 &
          length(unique(strsplit(gj,"")[[1]]))==2){out.msat <- r2*(1-rho2+r2)} else # all else, i.e. one mistype
        if((2-sum(unique(strsplit(kj,"")[[1]]) %in% unique(strsplit(gj,"")[[1]])))==2 &
          length(unique(strsplit(gj,"")[[1]]))==2){out.msat <- 2*r2^2} else # two mistypes, different allele manifests
        if((2-sum(unique(strsplit(kj,"")[[1]]) %in% unique(strsplit(gj,"")[[1]])))==2 &
          length(unique(strsplit(gj,"")[[1]]))==1){out.msat <- 1*r2^2} # two mistypes, same allele manifests
        } # end else for heterozygote latent genotype
      return(out.msat)
      } # end P.error.msat

  # Example: locus with 10 alleles, observed aA versus true Ab (i.e. latent heterozygote, single mistype)
  # with 5% per-LOCUS (translated to per-allele within call) allelic dropout and 2% per-LOCUS false allele
  # genotyping error rate:
    (P.error.msat(rho1=1 - sqrt(1-0.05), rho2=1 - sqrt(1-0.02), a=10, gj="aA", kj="Ab"))
    (P.error.msat(rho1=1 - sqrt(1-0.05), rho2=1 - sqrt(1-0.02), a=10, gj="Aa", kj="bA")) # allele order doesn't matter

#---------------------------------------------------------------------------------------------------------------#
# User defined functions to calculate probabilities of observing a pair of latent diploid genotypes k_1,k_2
# at locus j, given a proposed relationship state (R), Pr(k_1,k_2|R). These are intermediary to joint.prob.multi() below.
  # Inputs: vectors of allele frequencies, f; note, for probability calculations the order of allele
  # frequencies passed to a function matters (see comments below). Naming convention of functions indicate
  # the homozygous/heterozygous status of the pair of genotypes, the number of alleles in common, and the
  # proposed relationship state: Same Individual, Unrelated, Parent Offspring, or Full Sibling.  Probability formulae
  # are taken from Weir et al. (2006).

  # Needed for both biallelic SNPs and MSAT loci with >= 2 alleles at a locus:
    # Two homozygous genotypes, two shared alleles
    # Arguments: a single allele frequency corresponds to the matching allele
      hom.hom2.SI <- function(f){
        return(f[1]^2)
        } # End joint genotype probability calculation for observing ii/ii given same individual
      hom.hom2.U <- function(f){
        return(f[1]^4)
        } # End joint genotype probability calculation for observing ii/ii given two unrelated individuals
      hom.hom2.PO <- function(f){
        return(f[1]^3)
        } # End joint genotype probability calculation for observing ii/ii given a parent offspring relationship
      hom.hom2.FS <- function(f){
        return(0.25*f[1]^2 + 0.5*f[1]^3 + 0.25 * f[1]^4)
        } # End joint genotype probability calculation for observing ii/ii given two full siblings

    # Two homozygous genotypes, zero shared alleles
```

```
  # Arguments: vector of two allele frequencies which correspond to one homozygote and second to the
  # other, order doesn't matter
    hom.hom0.SI <- function(f){
      return(0)
      } # End joint genotype probability calculation for observing ii/jj given same individual
    hom.hom0.U <- function(f){
      return(f[1]^2 * f[2]^2)
      } # End joint genotype probability calculation for observing ii/jj given two unrelated individuals
    hom.hom0.PO <- function(f){
      return(0)
      } # End joint genotype probability calculation for observing ii/jj given a parent offspring relationship
    hom.hom0.FS <- function(f){
      return(0.25*f[1]^2 * f[2]^2)
      } # End joint genotype probability calculation for observing ii/jj given two full siblings

  # One homozygote, one heterozygote, one shared allele
  # Arguments: vector of two allele frequencies, here the first allele frequency must correspond to the
  # homozygous genotype and the second to the novel allele in the heterzygote, e.g. ii/ij => f = c(f_i,f_j).
    hom.het1.SI <- function(f){
      return(0)
      } # End joint genotype probability calculation for observing ii/ij given same individual
    hom.het1.U <- function(f){
      return(2*f[1]^3 * f[2])
      } # End joint genotype probability calculation for observing ii/ij given two unrelated individuals
    hom.het1.PO <- function(f){
      return(f[1]^2 * f[2])
      } # End joint genotype probability calculation for observing ii/ij given a parent offspring relationship
    hom.het1.FS <- function(f){
      return(0.5*f[1]^2 * f[2] + 2*0.25*f[1]^3 * f[2])
      } # End joint genotype probability calculation for observing ii/ij given two full siblings

  # Two heterozygote genotypes, two shared alleles
  # Arguments: a vector of two allele frequencies, the first allele corresponds to one allele in the
  # heterozygote, and the second the other, order doesn't matter
    het.het2.SI <- function(f){
      return(2*f[1] * f[2])
      } # End joint genotype probability calculation for observing ij/ij given same individual
    het.het2.U <- function(f){
      return(4*f[1]^2 * f[2]^2)
      } # End joint genotype probability calculation for observing ij/ij given two unrelated individuals
    het.het2.PO <- function(f){
      return(f[1] * f[2] * (f[1] + f[2]) )
      } # End joint genotype probability calculation for observing ij/ij given a parent offspring relationship
    het.het2.FS <- function(f){
      return( (2*0.25*f[1] * f[2]) + (0.5*f[1] * f[2] * (f[1] + f[2]) ) + (4*0.25*f[1]^2 * f[2]^2))
      } # End joint genotype probability calculation for observing ij/ij given two full siblings

# Needed only for multiallelic loci (e.g. MSATs) with > 2 alleles at a locus
  # One homozygote and one heterozygote, zero alleles shared
  # Arguments: a vector of three allele frequencies, the first allele frequency must correspond to the homozygous
  # genotype and the second and third to the novel alleles in the heterzygote, e.g. ii/jm => f = c(f_i,f_j,f_m)
    hom.het0.SI <- function(f){
      return(0)
      } # End joint genotype probability calculation for observing ii/jm given same individual
    hom.het0.U <- function(f){
      return(2*f[1]^2 * f[2] * f[3])
      } # End joint genotype probability calculation for observing ii/jm  given two unrelated individuals
```

```
      hom.het0.PO <- function(f){
        return(0)
        } # End joint genotype probability calculation for observing ii/jm  given a parent offspring relationship
      hom.het0.FS <- function(f){
        return(2*0.25*f[1]^2 * f[2] * f[3])
        } # End joint genotype probability calculation for observing ii/jm  given two full siblings

    # Two heterozygote genotypes, one allele shared
    # Arguments: a vector of three allele freqeuncies, the first allele corresponds to the common allele,
    # the second allele to the unique allele in k1, and the third allele frequency to the uniqe allele in k2,
    # e.g. for ij/im,  f = c(f_i,f_j,f_m)
      het.het1.SI <- function(f){
        return(0)
        } # End joint genotype probability calculation for observing ij/im given same individual
      het.het1.U <- function(f){
        return(4*1*f[1]^2 * f[2] * f[3])
        } # End joint genotype probability calculation for observing ij/im given two unrelated individuals
      het.het1.PO <- function(f){
        return(1*f[1] * f[2] * f[3] )
        } # End joint genotype probability calculation for observing ij/im given a parent offspring relationship
      het.het1.FS <- function(f){
        return(0.5*f[1] * f[2] * f[3] + 4*0.25*f[1]^2 * f[2] * f[3])
        } # End joint genotype probability calculation for observing ij/im given two full siblings

    # Two heterozygote genotypes, zero alleles shared
    # Here 0 alleles shared in common amongst pair of genotypes
    # Arguments: a vector of four allele frequencies; the allele frequency order matches that from each of the four
    # unique alleles across the pair of genotypes, e.g. for ij/ml, f=c(i,j,m,l)
      het.het0.SI <- function(f){
        return(0)
        } # End joint genotype probability calculation for observing ij/ml given same individual
      het.het0.U <- function(f){
        return(4*1*f[1] * f[2] * f[3] * f[4])
        } # End joint genotype probability calculation for observing ij/ml given two unrelated individuals
      het.het0.PO <- function(f){
        return(0)
        } # End joint genotype probability calculation for observing ij/ml given a parent offspring relationship
      het.het0.FS <- function(f){
        return(4*0.25*f[1] * f[2] * f[3] * f[4])
        } # End joint genotype probability calculation for observing ij/ml given two full siblings

#----------------------------------------------------------------------------------------------------------#
# User defined function for intermediary calculations of Pr(k1,k2|R) needed later to caculate the likelihood of a hypothesized
# relationship state between a pair of observed multilocus genotypes.

  # joint.prob.multi() -- This function outputs a dataframe whereby each row defines a proposed pair of diploid genotypes
  # at a locus, and the probability of observing this pair given a true relationship from which the pair of genotypes
  # originated, of Same Individual, Unrelated individuals, Parent Offspring, or Full Sibling.
  # Arguments: f. is a 1-row numeric object (works with 1-row matrix object or 1-row data.frame object with column
  # labels) containing allele frequencies for all alleles at a given locus, and with column
  # names corresponding to unique allele names as single character strings (e.g. lowercase letters).
  # Please code MSAT loci with many alleles carefully. Coding alleles as lower and upper case letters
  # and single numeric digits 0-9, can accommodate loci with 26+26+10 = 62 alleles, and could be expanded with other special
  # characters beyond that (e.g. "!" or "@"). Column naming corresponding to unique allele codes is needed to reference
  # the correct order of alleles when making probability calculations. Utilizes user defined functions for Pr(k_1,k_2|R)
  # above.
```

```
joint.prob.multi <- function(f.){
  # The total number of unique unordered pairs from a set of n unique elements is: n + choose(n,2) = n + (n!) / (2!(n-2)!)
    G.ord <- combn(x=rep(colnames(f.),2),m=2) # this creates all possible ORDERED diploid genotypes combinations with replacement
    G.unord <- unique(names(table(apply(G.ord,MARGIN=2,FUN=function(x){paste(sort(x),collapse="")})))) # get unique set of unordered pairs
  # A total of, length(G.unord)^2 possible ordered genotype 'dyads' of unique unordered diploid genotypes, with formatting into
  # a dataframe of four columns for four proposed relationship states:
    out <- data.frame(
      k1=rep(G.unord,each=length(G.unord)),
      k2=rep(G.unord,length(G.unord)),
      prob.SI=NA,prob.U=NA,prob.PO=NA,prob.FS=NA)
  # Now set up a series of genotype comparison challenges to come up with appropriate pairwise probability calculation.
  # There are seven total possible allele sharing outcomes to test.
    for(i in 1:nrow(out)){
      # break out each diploid genotype into component alleles for subsequent allele frequency referencing
        g1 <- strsplit(as.character(out$k1[i]), "")[[1]]
        g2 <- strsplit(as.character(out$k2[i]), "")[[1]]
      # hom.hom, 2 shared alleles
        if(length(unique(g1))==1 & length(unique(g2))==1 & length(table(c(g1,g2)))==1){
          out$prob.SI[i] <- hom.hom2.SI(f=f.[1,g1[1]])
          out$prob.U[i] <- hom.hom2.U(f=f.[1,g1[1]])
          out$prob.PO[i] <- hom.hom2.PO(f=f.[1,g1[1]])
          out$prob.FS[i] <- hom.hom2.FS(f=f.[1,g1[1]])
          } else
      # hom.hom, 0 shared alleles
        if(length(unique(g1))==1 & length(unique(g2))==1 & length(table(c(g1,g2)))==2){
          out$prob.SI[i] <- hom.hom0.SI(f=c(f.[1,g1[1]],f.[1,g2[1]]))
          out$prob.U[i] <- hom.hom0.U(f=c(f.[1,g1[1]],f.[1,g2[1]]))
          out$prob.PO[i] <- hom.hom0.PO(f=c(f.[1,g1[1]],f.[1,g2[1]]))
          out$prob.FS[i] <- hom.hom0.FS(f=c(f.[1,g1[1]],f.[1,g2[1]]))
          } else
      # hom.het, 1 shared alleles
        if(length(unique(g1))==1 & length(unique(g2))==2 & length(intersect(g1,g2))==1 |
          length(unique(g1))==2 & length(unique(g2))==1 & length(intersect(g1,g2))==1 ){
          out$prob.SI[i] <- hom.het1.SI(f=f.[1,names(sort(table(c(g1,g2)),decreasing=T))])
          out$prob.U[i] <- hom.het1.U(f=f.[1,names(sort(table(c(g1,g2)),decreasing=T))])
          out$prob.PO[i] <- hom.het1.PO(f=f.[1,names(sort(table(c(g1,g2)),decreasing=T))])
          out$prob.FS[i] <- hom.het1.FS(f=f.[1,names(sort(table(c(g1,g2)),decreasing=T))])
          } else
      # hom.het, 0 shared alleles
        if(length(unique(g1))==1 & length(unique(g2))==2 & length(intersect(g1,g2))==0 |
          length(unique(g1))==2 & length(unique(g2))==1 & length(intersect(g1,g2))==0 ){
          out$prob.SI[i] <- hom.het0.SI(f=f.[1,names(sort(table(c(g1,g2)),decreasing=T))])
          out$prob.U[i] <- hom.het0.U(f=f.[1,names(sort(table(c(g1,g2)),decreasing=T))])
          out$prob.PO[i] <- hom.het0.PO(f=f.[1,names(sort(table(c(g1,g2)),decreasing=T))])
          out$prob.FS[i] <- hom.het0.FS(f=f.[1,names(sort(table(c(g1,g2)),decreasing=T))])
          } else
      # het.het, 2 shared alleles
        if(length(unique(g1))==2 & length(unique(g2))==2 & length(intersect(g1,g2))==2){
          out$prob.SI[i] <- het.het2.SI(f=f.[1,names(sort(table(c(g1,g2)),decreasing=T))])
          out$prob.U[i] <- het.het2.U(f=f.[1,names(sort(table(c(g1,g2)),decreasing=T))])
          out$prob.PO[i] <- het.het2.PO(f=f.[1,names(sort(table(c(g1,g2)),decreasing=T))])
          out$prob.FS[i] <- het.het2.FS(f=f.[1,names(sort(table(c(g1,g2)),decreasing=T))])
          } else
      # het.het, 1 shared alleles
        if(length(unique(g1))==2 & length(unique(g2))==2 & length(intersect(g1,g2))==1){
          out$prob.SI[i] <- het.het1.SI(f=f.[1,names(sort(table(c(g1,g2)),decreasing=T))])
          out$prob.U[i] <- het.het1.U(f=f.[1,names(sort(table(c(g1,g2)),decreasing=T))])
```

```
          out$prob.PO[i] <- het.het1.PO(f=f.[1,names(sort(table(c(g1,g2)),decreasing=T))])
          out$prob.FS[i] <- het.het1.FS(f=f.[1,names(sort(table(c(g1,g2)),decreasing=T))])
          } else
      # het.het, 0 shared alleles
        if(length(unique(g1))==2 & length(unique(g2))==2 & length(intersect(g1,g2))==0){
          out$prob.SI[i] <- het.het0.SI(f=f.[1,names(sort(table(c(g1,g2)),decreasing=T))])
          out$prob.U[i] <- het.het0.U(f=f.[1,names(sort(table(c(g1,g2)),decreasing=T))])
          out$prob.PO[i] <- het.het0.PO(f=f.[1,names(sort(table(c(g1,g2)),decreasing=T))])
          out$prob.FS[i] <- het.het0.FS(f=f.[1,names(sort(table(c(g1,g2)),decreasing=T))])
          }
      } # end i loop over rows of out matrix
    return(out)
  } # End joint.prob.multi function

  # Examples:
    # 5-allele locus, equal allele frequencies
      n.allele <- 5
      f.df <- matrix(nr=1,nc=length(letters[1:n.allele]),rep(1/n.allele,n.allele))
      colnames(f.df) <- letters[1:n.allele]
      (joint.prob.multi(f.=f.df))

### Section 2: Example simulated data ##############################################################################

 #---------------------------------------------------------------------------------------------------------------#
 # Simulate diploid "MSAT" type genotype data with potential allelic dropout and mistyping genotyping error.

 # MSAT.G.simulator() -- this function produces diploid multilocus genotypes from unrelated individuals
 # under a specified set of allele frequencies and genotyping error rates.  A list of objects is
 # returned: the simulated genotypes with a leading column indexing a generic sampling id, and a last column
 # indicating true individual id, followed by all other inputs passed back to the user.
 # Arguments: F.sim.ls = a list object where each element is a 1 row matrix of
 # allele frequencies and with  column names indicating allele names (must be single-character strings, e.g.
 # lower case letters or upper case letters) with one row for each locus simulated. Thus, the number of elements
 # in the F.sim.ls sets the number of loci. See example below; ADO.locus = vector of locus-level
 # (vs. per-allele rate) allelic dropout rates; FA.locus = vector of locus-level (vs. per-allele rate)
 # mistype rates; n.unique = total number of unique individuals for which to simualte genotypes;
 # id.start = integer at which unique individual id numbering commences; recap.ix = vector indicating which
 # of 1:n.unique individuals is recaught, which can include repeated captures of individuals (e.g.
 # recap.ix = c(1,1,2,3)).  If left as 'NA', no recaptures are simulated; r.seed = a random number seed.
 # If left as 'NA', then no seed is passed.  Note, allelic dropout rates are relevant to true latent
 # heterozygotes; false allele rates are relevant to both latent heterozygotes and homozygotes,
 # e.g. see Broquet and Petit (2004).

 MSAT.G.simulator <- function(F.sim.ls,ADO.locus,FA.locus,n.unique,id.start,recap.ix=NA,r.seed=NA){
   # Set seed
     if(is.na(r.seed)==F){set.seed(r.seed)}
   # Translate locus-level error rate to per-allele rates (same for all loci here)
     # ADO
     err.ado <- 1-sqrt(1-ADO.locus)
     # FA
     err.fa <- 1-sqrt(1-FA.locus)
   # Calculate number recaptures
     if(length(recap.ix)==1 & sum(is.na(recap.ix))==1) {n.recap <- 0} else {n.recap <- length(recap.ix)}
   # Declare indexing varibles
     n.loci <- length(F.sim.ls)
     col.even <- seq(from=2,to=2*n.loci,by=2)
     col.odd <- seq(from=1,to=2*n.loci,by=2)
```

```
  # Set up genotype storage object
    G.lat <- data.frame(matrix(nr=n.unique+n.recap,nc=2*n.loci))
  # True genotypes for n.unique individuals
    for(j in 1:n.loci){
      G.lat[1:n.unique,col.odd[j]] <- sample(colnames(F.sim.ls[[j]]),size=n.unique,prob=F.sim.ls[[j]],replace=T)
      G.lat[1:n.unique,col.even[j]] <- sample(colnames(F.sim.ls[[j]]),size=n.unique,prob=F.sim.ls[[j]],replace=T)
      } # end j loop over loci
  # Simulate recaptured samples and populate sample and true identifications
    if(n.recap>0) {G.lat[(n.unique+1):(n.unique+n.recap),] <- G.lat[recap.ix,]}
  # Generate observed genotypes, following the two-class genotyping error (allelic dropout and false allele)
  # model as specified in Wang (2004, 2006, 2016)
    G.obs <- G.lat
    for(j in 1:n.loci){
      # ADO happens first. Following Wang (2004, 2016) ADO model, then no ADO = 1-2*(err.ado[j]/(1+err.ado[j]))
      # and thus prob. dropout is 1-(1-2*(err.ado[j]/(1+err.ado[j]))) = 2*(err.ado[j]/(1+err.ado[j]))
      # Equal chance of allele 1 or 2 dropping out, so for ease, just use first allele if ADO occurs
        temp.err <- rbinom(n=nrow(G.lat),size=1,prob=2*(err.ado[j]/(1+err.ado[j])))
        if(sum(temp.err)>0){G.obs[temp.err==1,col.odd[j]:col.even[j]] <- G.obs[temp.err==1,col.odd[j]]}
      # FA happens second, and 0,1, or 2 alleles can mistype.
        # first allele copy for locus j, across all rows of G matrix
          temp.err <- rbinom(n=nrow(G.obs),size=1,prob=err.fa[j])
          if(sum(temp.err)>0){
          G.obs[temp.err==1,col.odd[j]] <- sapply(G.obs[temp.err==1,col.odd[j]],FUN=function(x){
            sample(colnames(F.sim.ls[[j]])[colnames(F.sim.ls[[j]])!=x],size=1,prob=F.sim.ls[[j]][colnames(F.sim.ls[[j]])!=x],
            replace=T)})}
        # second allele copy for locus j, across all rows of G matrix
          temp.err <- rbinom(n=nrow(G.lat),size=1,prob=err.fa[j])
          if(sum(temp.err)>0){
          G.obs[temp.err==1,col.even[j]] <- sapply(G.obs[temp.err==1,col.even[j]],FUN=function(x){
            sample(colnames(F.sim.ls[[j]])[colnames(F.sim.ls[[j]])!=x],size=1,prob=F.sim.ls[[j]][colnames(F.sim.ls[[j]])!=x],
            replace=T)})}
      } # end j loop over loci for genotyping error
  # Add in columns for sample vs. true identification
    if(n.recap>0){G.obs <- cbind(id.start:(id.start-1 + n.unique + n.recap),G.obs,
      c(id.start:(id.start-1+n.unique),(id.start:(id.start-1 + n.unique))[recap.ix]))} else
      {G.obs <- cbind(id.start:(id.start-1 + n.unique),G.obs,id.start:(id.start-1 + n.unique))}
    colnames(G.obs)[c(1,ncol(G.obs))] <- c("Sample.ID","True.ID")
    return(structure(list(G.obs,F.sim.ls,ADO.locus,FA.locus,n.unique,recap.ix,r.seed),
      .Names=c("G.obs","F.sim.ls","ADO.locus","FA.locus","n.unique","recap.ix","r.seed")))
    } # end function MSAT.G.simulator()

# Examples:
# Generate an example data set for stage-1 clustering with which to identify within-sampling occasion recaptures.
# 10 "MSAT" loci each with 5 equal frequency alleles, a 5% allelic dropout error rate and 2% locus-level false
# allele rate. Generate multilocus genotypes for 15 unique unrelated individuals, a double recapture of individual 1,
# and single recaptures of individuals 2-4.
# Simulation parameters:
  num.loci=10; num.allele=5; allele.names=letters[1:num.allele]
  F.ls <- lapply(1:num.loci,FUN=function(j){
            x <- t(as.matrix(rep(1/num.allele,num.allele)));
            colnames(x) <- allele.names;
            return(x)})
  Ss1.sim <- MSAT.G.simulator(F.sim.ls=F.ls, ADO.locus = rep(0.05,num.loci), FA.locus = rep(0.02,num.loci),
    n.unique = 15, id.start=100,recap.ix = c(1,1,2,3,4),r.seed = 1)

# Generate a pair of genotype data sets for testing of stage-2 sample clustering to identify between-sampling occasion
# recaptures.  Set 1, as above but made up of 20 unique individuals.  Set 2 made up of 5 recaptures from Set 1, and
```

```
  # 15 unique unrelated individuals.
    Ss2_1.sim <- MSAT.G.simulator(F.sim.ls=F.ls, ADO.locus = rep(0.05,num.loci), FA.locus = rep(0.02,num.loci),
      n.unique = 20, id.start=100,recap.ix = NA,r.seed = 2)
    Ss2_2.sim <- MSAT.G.simulator(F.sim.ls=F.ls, ADO.locus = rep(0.05,num.loci), FA.locus = rep(0.02,num.loci),
      n.unique = 15, id.start=200,recap.ix = NA,r.seed = 3)
    Ss2_2.sim$G.obs <- rbind(Ss2_2.sim$G.obs,Ss2_1.sim$G.obs[1:5,]) # create recaptures in sampling occasion 2
    # repopulate a generic sample ID number for occasion 2
    Ss2_2.sim$G.obs$Sample.ID <- Ss2_2.sim$G.obs$Sample.ID[1]:(Ss2_2.sim$G.obs$Sample.ID[1] + nrow(Ss2_2.sim$G.obs)-1)


### Section 3: Stage-one clustering to identify repeated captures within a single sampling occasion ############################
#--------------------------------------------------------------------------------------------------------------#
# Inputs
  # Get genotype data from a single capture occasion.
  # NOTE: Input genotype data need have in column 1 a sample ID, followed by a column for
  # each allele copy for each diploid locus.  Allele calls need be single character strings.
  # Please code MSAT loci with many alleles carefully. Coding alleles as lower and upper case
  # letters and single numeric digits 0-9, can accommodate loci with 26+26+10 = 62 alleles, and could be
  # expanded further if need be with other special characters read in as strings beyond that (e.g. "!" or "@").
  # See example output from MSAT.G.simulator() for input genotype formatting examples.
    S.input <- Ss1.sim$G.obs[,1:(ncol(Ss1.sim$G.obs)-1)] # Don't need the true individual ID column
  # Get allele frequency matrix.  Format: a list object where each element is a 1 row matrix of allele
  # frequencies and with  column names indicating allele nameswith one row for each locus simulated. Thus,
  # the number of elements in the F.sim.ls sets the number of loci.
    F.m <- Ss1.sim$F.sim.ls # F.sim.m
  # Declare vectors of locus-level error rates
    # ADO
    err.ado.l <- Ss1.sim$ADO.locus
    # FA
    err.fa.l <- Ss1.sim$FA.locus

#--------------------------------------------------------------------------------------------------------------#
# Initialize intermediate quantities and storage objects
  # Get number of alleles at each locus
    n.allele <- sapply(F.m,FUN=function(x){length(x)})
  # Get number of loci
    n.loci <- length(F.m)
  # Convert locus-level error rates to per-allele rates
    # ADO
      err.ado <- 1-sqrt(1-err.ado.l)
    # FA
      err.fa <- 1-sqrt(1-err.fa.l)

  # Given the input allele frequencies, define joint probabilities given different relationship states,
  # using the joint.prob.multi() user defined function. Can take time to generate if many alleles at loci.
  # The idea is to calculate this once, and then reference relevant genotype pairs during the clustering
  # loop later.
    joint.prob.ls <- list()
    for(L in 1:length(F.m)){
      joint.prob.ls[[L]] <- joint.prob.multi(f.=F.m[[L]])
      }
  # Column indexing vector--be careful here, this relies on specific format of input genotypes (see above).
    col.even <- seq(from=2,to=ncol(S.input)-1,by=2)
    col.odd <- seq(from=3,to=ncol(S.input),by=2)
  # Initialize storage variables, helper functions
    n.prob.ls <- sapply(joint.prob.ls,FUN=function(x){nrow(x)})
  # Helper function
```

```
    `%notin%`<- Negate(`%in%`)
  # Storage matrix for L(observed genotypes in pair of sets | relationship) at each locus, j.
  # A matrix of dimension rows = # loci, cols = 4 (in order, SI, U, PO, FS)
    LRG1G2j <- matrix(nr=n.loci,nc=4,1)
    colnames(LRG1G2j) <- c("SI","U","PO","FS") # assign colnames to object for record keeping/debugging
  # Storage for Likelihood product across all (assumed independent) loci
    LRG1G2 <- 1:4 # L| SI, U, PO, FS

#---------------------------------------------------------------------------------------------------------------#
# Stage-one clustering in action. This code contains several error traps; see code comments below.
  # Step 1: Intialize the clustering algorithm with all samples as singleton sets, a list object with sample IDs.
    S <- list()
    for(z in 1:nrow(S.input)){S[[z]] <- S.input[z,1]} # sample ID taken from first column
    S.old <- S # temporary copy
  # Steps 2-3: Pairwise comparisons for match calls, repeating until set membership stops changing
     repeat{
      S.old <- S
      for(i in 1:length(S)){
        # Likelihood ratio and set membership sample ID labels placeholders; need list objects here s.t.
        # a given element can hold more than one multilocus genotype if need be.
          temp.rat.sc <- list()
          temp.rat.mr <- list()
        # Indexing variable place holder
          ix1 <- 0
        # Exit trap, don't make a match comparison if S[[i]] set is NA, i.e. G1 is empty set
          if(sum(is.na(S[[i]]))>0) {next}
        # Indexing construct for i and k>i results in all unique pairwise comparisons across sets
          for(k in (1:length(S))[(1:length(S))>i]){
            # Get the pair of sets of genotypes to compare
              G1 <- S.input[S.input[,1]%in%S[[i]],]
              G2 <- S.input[S.input[,1]%in%S[[k]],]
            # Exit trap, if G2 is empty set proceed to next k
              if(nrow(G2)==0) {next}
            # Determine which are common positive PCR loci, i.e. the set of loci for which both samples
            # have any genotype call. Note, the below code still works when a given G1 or G2 set has multiple
            # elements.
              pos.loci.ix <- col.even[(nrow(G1)-colSums(is.na(G1[col.even])))>0 &
                (nrow(G2)-colSums(is.na(G2[col.even])))>0]/2
            # Update indexing variable
              ix1 <- ix1+1
            # Exit trap, if pos.loci.ix is zero, implying no common loci with genotypes which can happen with real-world
            # data with failed PCR outcomes across some loci.  One could impose a common-loci minimum threshold here
            # as well. Note, exit in this case results in retaining separation of the two compared sample sets, implying
            # they derive from separate individuals.
              if(length(pos.loci.ix)==0) {
                # store index information for compared sets, and evidence ratio
                temp.rat.sc[[ix1]] <- G2[,1]
                temp.rat.mr[[ix1]] <- 0 # force to zero
                next
                } # end if
            # Make calculations for likelihood across loci, Lj(R_(1,2)), for each locus
            for(j in 1:n.loci){
              # First, determine whether there is missing genotype info among the two compared sets at this locus,
              # using pos.loci.ix from above.  If yes, pass a 1.0 value which will not affect likelihood
              # multiplication subsequently (i.e. Like * 1 = Like).
                if(j %notin% pos.loci.ix){
                  LRG1G2j[j,1:4] <- rep(1,4)
```

```
          next} # end if; 'next' goes to next locus
      # Reference the set of possible pairs of genotypes at a given locus and associated joint probabilities
      # calculated with joint.prob.multi() outside the clustering algorithm, Pr(kj1,kj2|R),
        temp.prob <- joint.prob.ls[[j]]
      # Calculate the probability of observed genotypes given proposed latent genotypes, Pr(gj|kj).
      # This code accomodates multiple genotypes within a set, e.g. if after an
      # iteration of the clustering algorithm, it is found that two samples from a single individual are
      # grouped into one set.
        # Storage vector, defined relevant to number of alleles at locus j
          Pg1_k1 <- rep(1,n.prob.ls[[j]])
          Pg2_k2 <- Pg1_k1
        for(ii in 1:nrow(G1)){
          # First, determine whether there is a missing locus; if yes, then exit
            if(sum(is.na(G1[ii,(col.even[j]:col.odd[j])]))>0){next}
          # Else, populate Pr(gj|kj) for all possible kj
            Pg1_k1[1:n.prob.ls[[j]]] <- Pg1_k1 * sapply(temp.prob$k1, FUN=function(x){
              P.error.msat(rho1=err.ado[j],rho2=err.fa[j],a=n.allele[j],
              gj=paste(G1[ii,(col.even[j]:col.odd[j])],collapse=""),kj=as.character(x))})
          }
        for(ii in 1:nrow(G2)){
            if(sum(is.na(G2[ii,(col.even[j]:col.odd[j])]))>0){next}
            Pg2_k2[1:n.prob.ls[[j]]] <- Pg2_k2 * sapply(temp.prob$k2, FUN=function(x){
              P.error.msat(rho1=err.ado[j],rho2=err.fa[j],a=n.allele[j],
              gj=paste(G2[ii,(col.even[j]:col.odd[j])],collapse=""),kj=as.character(x))})
          }
      # Crossproducts to sum over all combinations of Pr(k1k2|R) * Pr(g1|k1) * Pr(g2|k2) at this locus
        LRG1G2j[j,1] <- as.numeric(temp.prob$prob.SI) %*% (Pg1_k1*Pg2_k2)
        LRG1G2j[j,2] <- as.numeric(temp.prob$prob.U) %*% (Pg1_k1*Pg2_k2)
        LRG1G2j[j,3] <- as.numeric(temp.prob$prob.PO) %*% (Pg1_k1*Pg2_k2)
        LRG1G2j[j,4] <- as.numeric(temp.prob$prob.FS) %*% (Pg1_k1*Pg2_k2)
      } # end j loop over loci
  # Now product across loci, L(R_(1,2))
    LRG1G2 <- apply(LRG1G2j,MARGIN=2,FUN=prod)
  # Store index information for compared sets, and likelihood ratios of L(R_(1,2)=SI)/L(R_(1,2)=other).
  # See Wang(2004,2006,2016), Kalinowski et al. (2006), and Supplement 1 to this article for details
  # on the sample matching likelihood ratio model.
    temp.rat.sc[[ix1]] <- G2[,1]
    temp.rat.mr[[ix1]] <- LRG1G2[1]/max(LRG1G2[2:4],na.rm=T)
    } # end k loop over all samples > i (pairwise combos)
# Update set membership by combining G2 into G1 if max likelihood ratio > 1.0
  if(length(temp.rat.mr)>0){
    # As written here, temp.Sample.Code can be multiple different sets if more than one
    # set produces a max evidence ratio
      temp.rat.mr.v <- unlist(temp.rat.mr)
      temp.Sample.Code <-
        unlist(temp.rat.sc[temp.rat.mr.v==max(temp.rat.mr.v,na.rm=T) & temp.rat.mr.v>1.0])
        } else {temp.Sample.Code <- NULL}
      S[[i]] <- c(S[[i]],temp.Sample.Code)
  # Now  remove the sample codes from the singleton sets such that it isn't considered for
  # allocation again later
    if(length(temp.Sample.Code)>0){
      S[unlist( lapply(S,FUN=function(x){sum(x%in%temp.Sample.Code)==length(x)}) )] <- NA
      } # end if
  } # end i loop
S <- lapply(S, function(x) x[!is.na(x)]) # Cleaning up
S <- S[lapply(S,length)>0] # Cleaning up
if(identical(S,S.old)==TRUE){break}
```

```
      } # End repeat when no new updating, step 4.

      # Convert the final S list to a matrix object to examine clustering
        S.m <- matrix(nr=length(S),nc=max(unlist(lapply(S,FUN=length))),NA )
        for(r in 1:nrow(S.m)){
          S.m[r,1:length(S[[r]])] <- S[[r]]
          } # end r loop
        # print
          S.m # Unique individuals along rows, any corresponding recaptures' Sample IDs in column 2+
          Ss1.sim$G.obs[,c("Sample.ID","True.ID")] # Cross reference Sample IDs to verify recapture identity

### Section 4: Stage two clustering to identify recaptures across sampling occasions ################################
#-------------------------------------------------------------------------------------------------------------#
# Inputs
  # Get genotype data from a pair of capture occasions.
  # NOTE: Input genotype data need have in column 1 a sample ID, followed by a column for
  # each allele copy for each diploid locus.  Allele calls need be single character strings.
  # Please code MSAT loci with many alleles carefully. Coding alleles as lower and upper case
  # letters and single numeric digits 0-9, can accommodate loci with 26+26+10 = 62 alleles, and could be
  # expanded further if need be with other special characters read in as strings beyond that (e.g. "!" or "@").
  # See example output from MSAT.G.simulator() for input genotype formatting examples.
    S1.input <- Ss2_1.sim$G.obs[,1:(ncol(Ss2_1.sim$G.obs)-1)] # Don't need the true individual ID column
    S2.input <- Ss2_2.sim$G.obs[,1:(ncol(Ss2_2.sim$G.obs)-1)] # Don't need the true individual ID column
  # Get allele frequency matrix.  Format: a list object where each element is a 1 row matrix of allele
  # frequencies and with  column names indicating allele nameswith one row for each locus simulated. Thus,
  # the number of elements in the F.sim.ls sets the number of loci.
    F.m <- Ss1.sim$F.sim.ls # F.sim.m
  # Declare vectors of locus-level error rates
    # ADO
      err.ado.l <- Ss2_1.sim$ADO.locus
    # FA
      err.fa.l <- Ss2_1.sim$FA.locus

#-------------------------------------------------------------------------------------------------------------#
# Initialize intermediate quantities and storage objects
  # Get number of alleles at each locus
    n.allele <- sapply(F.m,FUN=function(x){length(x)})
  # Get number of loci
    n.loci <- length(F.m)
  # Convert locus-level error rates to per-allele rates
    # ADO
      err.ado <- 1-sqrt(1-err.ado.l)
    # FA
      err.fa <- 1-sqrt(1-err.fa.l)

  # Given the input allele frequencies, define joint probabilities given different relationship states,
  # using the joint.prob.multi() user defined function. Can take time to generate if many alleles at loci.
  # The idea is to calculate this once, and then reference relevant genotype pairs during the clustering
  # loop later.
    joint.prob.ls <- list()
    for(L in 1:length(F.m)){
      joint.prob.ls[[L]] <- joint.prob.multi(f.=F.m[[L]])
      }
  # Column indexing vector--be careful here, this relies on specific format of input genotypes (see above).
    col.even <- seq(from=2,to=ncol(S.input)-1,by=2)
    col.odd <- seq(from=3,to=ncol(S.input),by=2)
  # Initialize storage variables, helper functions
```

```
    n.prob.ls <- sapply(joint.prob.ls,FUN=function(x){nrow(x)})
  # Helper function
    `%notin%`<- Negate(`%in%`)
  # Storage matrix for L(observed genotypes in pair of sets | relationship) at each locus, j.
  # A matrix of dimension rows = # loci, cols = 4 (in order, SI, U, PO, FS)
    LRG1G2j <- matrix(nr=n.loci,nc=4,1) # SI, U, PO, FS is order
    colnames(LRG1G2j) <- c("SI","U","PO","FS") # assign colnames to object for record keeping/debugging
  # Storage for Likelihood product across all (assumed independent) loci
    LRG1G2 <- 1:4 # L| SI, U, PO, FS

#--------------------------------------------------------------------------------------------------------------#
# Stage-two clustering in action. This code contains several error traps; see code comments below.
  # Intialize the clustering algorithm with all samples as singleton sets, list objects with sample IDs.
    S1 <- list()
    S2 <- list()
    for(z in 1:nrow(S1.input)){S1[[z]] <- S1.input[z,1]} # sample ID is in first column
    for(z in 1:nrow(S2.input)){S2[[z]] <- S2.input[z,1]}
  # Steps 1-2: Pairwise comparisons for match calls
      for(i in 1:length(S1)){
        # Likelihood ratio and set membership sample ID labels placeholders; need list objects here s.t.
        # a given element can hold more than one multilocus genotype if need be.
          temp.rat.sc <- list()
          temp.rat.mr <- list()
        # Indexing variable place holder
          ix1 <- 0
        # Exit trap, don't make a match comparison if S1[[i]] set is NA, i.e. G1 is empty set
          if(sum(is.na(S1[[i]]))>0) {next}
        # Indexing across sampling occaions, compare each ith sample in S1 to all samples in S2 for recaptures
          for(k in 1:length(S2)){
            # Get the pair of sets of genotypes to compare
              G1 <- S1.input[S1.input[,1]%in%S1[[i]],]
              G2 <- S2.input[S2.input[,1]%in%S2[[k]],]
            # Exit trap, if G2 is empty set proceed to next k
              if(nrow(G2)==0) {next}
            # Determine which are common positive PCR loci, i.e. the set of loci for which both samples
            # have any genotype call. Note,the below code still works when a given G1 or G2 set has multiple
            # elements
              pos.loci.ix <- col.even[(nrow(G1)-colSums(is.na(G1[col.even])))>0 &
                (nrow(G2)-colSums(is.na(G2[col.even])))>0]/2
            # Update indexing variable
              ix1 <- ix1+1
            # Exit trap, if pos.loci.ix is zero, implying no common loci with genotypes which can happen with real-world
            # data with failed PCR outcomes across some loci.  One could impose a common-loci minimum threshold here
            # as well. Note, exit in this case results in retaining separation of the two compared sample sets, implying
            # they derive from separate individuals.
              if(length(pos.loci.ix)==0) {
                # store index information for compared sets, and evidence ratio
                temp.rat.sc[[ix1]] <- G2[,1]
                temp.rat.mr[[ix1]] <- 0 # force to zero
                next
                } # end if
            # Make calculations for likelihood across loci, Lj(R_(1,2)), for each locus
            for(j in 1:n.loci){
              # First, determine whether there is missing genotype info among the two compared sets at this locus,
              # using pos.loci.ix from above.  If yes, pass a 1.0 value which will not affect likelihood
              # multiplication subsequently (i.e. Like * 1 = Like).
                if(j %notin% pos.loci.ix){
```

```
          LRG1G2j[j,1:4] <- rep(1,4)
          next} # end if; 'next' goes to next locus
      # Reference the set of possible pairs of genotypes at a given locus and associated joint probabilities
      # calculated with joint.prob.multi() outside the clustering algorithm, Pr(kj1,kj2|R),
        temp.prob <- joint.prob.ls[[j]]
      # Calculate the probability of observed genotypes given proposed latent genotypes, Pr(gj|kj).
      # This code accomodates multiple genotypes within a set, e.g. if after an
      # iteration of the clustering algorithm, it is found that two samples from a single individual are
      # grouped into one set.
        # Storage vector, defined relevant to number of alleles at locus j
          Pg1_k1 <- rep(1,n.prob.ls[[j]])
          Pg2_k2 <- Pg1_k1
        for(ii in 1:nrow(G1)){
          # First, determine whether there is a missing loci; if yes, then exit
            if(sum(is.na(G1[ii,(col.even[j]:col.odd[j])]))>0){next}
          # Else, populate Pr(gj|kj) for all possible kj
            Pg1_k1[1:n.prob.ls[[j]]] <- Pg1_k1 * sapply(temp.prob$k1, FUN=function(x){
              P.error.msat(rho1=err.ado[j],rho2=err.fa[j],a=n.allele[j],
              gj=paste(G1[ii,(col.even[j]:col.odd[j])],collapse=""),kj=as.character(x))})
          }
        for(ii in 1:nrow(G2)){
            if(sum(is.na(G2[ii,(col.even[j]:col.odd[j])]))>0){next}
            Pg2_k2[1:n.prob.ls[[j]]] <- Pg2_k2 * sapply(temp.prob$k2, FUN=function(x){
              P.error.msat(rho1=err.ado[j],rho2=err.fa[j],a=n.allele[j],
              gj=paste(G2[ii,(col.even[j]:col.odd[j])],collapse=""),kj=as.character(x))})
          }
      # Crossproducts to sum over all combinations of Pr(k1k2|R) * Pr(g1|k1) * Pr(g2|k2) at this locus
        LRG1G2j[j,1] <- as.numeric(temp.prob$prob.SI) %*% (Pg1_k1*Pg2_k2)
        LRG1G2j[j,2] <- as.numeric(temp.prob$prob.U) %*% (Pg1_k1*Pg2_k2)
        LRG1G2j[j,3] <- as.numeric(temp.prob$prob.PO) %*% (Pg1_k1*Pg2_k2)
        LRG1G2j[j,4] <- as.numeric(temp.prob$prob.FS) %*% (Pg1_k1*Pg2_k2)
      } # end j loop over loci
  # Now product across loci, L(R_(1,2))
    LRG1G2 <- apply(LRG1G2j,MARGIN=2,FUN=prod)
  # Store index information for compared sets, and likelihood ratios of L(R_(1,2)=SI)/L(R_(1,2)=other).
  # See Wang(2004,2006,2016), Kalinowski et al. (2006), and Supplement 1 to this article for details
  # on the sample matching likelihood ratio model.
    temp.rat.sc[[ix1]] <- G2[,1]
    temp.rat.mr[[ix1]] <- LRG1G2[1]/max(LRG1G2[2:4],na.rm=T)
    } # end k loop over all samples > i (pairwise combos)
# Update set membership by combining G2 into G1 if max likelihood ratio > 1.0
  if(length(temp.rat.mr)>0){
    # As written here, temp.Sample.Code can be multiple different sets if more than one
    # set produces a max evidence ratio
      temp.rat.mr.v <- unlist(temp.rat.mr)
      temp.Sample.Code <-
        unlist(temp.rat.sc[temp.rat.mr.v==max(temp.rat.mr.v,na.rm=T) & temp.rat.mr.v>1.0])
        } else {temp.Sample.Code <- NULL}
      S1[[i]] <- c(S1[[i]],temp.Sample.Code)
  # Now  remove the sample codes from the singleton sets in S2 such that it isn't considered for
  # allocation again later
    if(length(temp.Sample.Code)>0){
      S2[unlist( lapply(S2,FUN=function(x){sum(x%in%temp.Sample.Code)==length(x)}) )] <- NA
      } # end if
  } # end i loop
S2 <- lapply(S2, function(x) x[!is.na(x)]) # Cleaning up
S2 <- S2[lapply(S2,length)>0] # Cleaning up
```

```
      # Convert the final S1 list to a matrix object to examine clustering.  Those rows (sets) with > 2 members
      # indicate the sample IDs captured in both occasion one and occasion
        S.m <- matrix(nr=length(S1),nc=max(unlist(lapply(S1,FUN=length))),NA )
        for(r in 1:nrow(S.m)){
          S.m[r,1:length(S1[[r]])] <- S1[[r]]
          } # end r loop
        # print
          S.m # Sample IDs from Set 1 in column 1 and any corresponding recaptures' Sample IDs in column 2
          Ss2_2.sim$G.obs[,c("Sample.ID","True.ID")] # Cross reference Sample IDs to verify recapture identity
```


```
### Section 5: References ###################################################################################################
  # Broquet T, Petit E. 2004 Quantifying genotyping errors in noninvasive population
    # genetics. Molecular Ecology 13, 3601-3608.
  # Kalinowski ST, Taper ML, Creel S. 2006 Using DNA from non-invasive
    # samples to identify individuals and census populations: an evidential
    # approach tolerant of genotpying errors. Conserv. Genet. 7, 319-329.
  # Wang J. 2004 Sibship reconstruction from genetic data with typing errors.
    # Genetics 166, 1963-1979.
  # Wang J. 2006 Informativeness of genetic markers for pairwise relationship
    # and relatedness inference. Theor. Popul. Biol. 70, 300-321.
  # Wang J. 2016 Individual identification from genetic marker data: developments
    # and accuracy comparisons of methods. Mol. Ecol. Resour. 16, 163-175.
  # Weir B, Anderson AD, Hepler AB. 2006 Genetic relatedness analysis: modern
    # data and new challenges. Nat. Rev. Genet. 7, 771-780.
```

Supplement 3: R script to implement the error-tolerant likelihood-based match calling model and sample clustering algorithms: SNPs

```
# Example code to conduct likelihood-based error-tolerant sample matching and clustering for
# genetic mark recapture studies: biallelic markers for diploid genotypes.

# Version 1.0-Nov09_2016

# Sethi SA, Linden D, Wenburg J, Lewis C, Lemons P, Fuller A, Hare M

# Overview: This script contains code to implement likelihood-based error-
# tolerant "SNP" genotype matching, and subsequent stage 1 (within a sampling occasion)
# and stage 2 (between sampling occasions) clustering to identify recaptures.
# See Supplement 1 of Sethi et al. for explanation of methods and description
# of formulae.  Below, references are provided indicating source articles from which
# probability models and genotyping error models were derived.
# The intent of this script is to provide example code
# with which to implement likelihood-based error-tolerant genotype matching
# and sample clustering for genetic mark recapture data, however this code is
# not maintained by the authors.

# Contents:
# Section 1: User defined functions
# Section 2: Example data simulation
# Section 3: Stage one clustering to identify repeated captures within a single sampling occasion
# Section 4: Stage two clustering to identify recaptures across sampling occasions
# Section 5: References

### Section 1: User defined functions (load these first) ####################################################################

  #-----------------------------------------------------------------------------------------------------------#
  # User defined function to calculate the probability of observing a sample genotype at a locus
  # given a proposed true latent genotype, Pr(g_ij|k_ij).

    # SNP genotyping error model
      # P.error.snp() -- Calculates the probability of observing a sample genotype at locus j given a true
      # latent genotype k for biallelic SNP markers.
      # Arguments: gamma_j = per-allele SNP genotyping error rate at locus j; n.a = total number of alleles
      # assessed for discrepancies (i.e. for biallelic SNPs this is 2*number of genotypes assessed at locus j);
      # d_gk = number of alleles discrepant between observed genotype g and latent genotype k in assessing
      # n.a total alleles
        P.error.snp <- function(gamma_j, d_gk, n.a){
          return( (gamma_j)^(d_gk) * (1-gamma_j)^(n.a-d_gk) )
          }
      # Example: observed aA versus true AA with 5% per-LOCUS genotyping error rate:
        (P.error.snp(gamma_j = 1 - sqrt(1-0.05), n.a = 2*1, d_gk = 1))
      # Example: observed {aA,aa} versus true AA with 5% per-LOCUS genotyping error rate:
        (P.error.snp(gamma_j = 1 - sqrt(1-0.05), n.a = 2*2, d_gk = 3))

  #-----------------------------------------------------------------------------------------------------------#
  # User defined functions to calculate probabilities of observing a pair of latent diploid genotypes k_1,k_2
  # at locus j, given a proposed relationship state (R), Pr(k_1,k_2|R). These are intermidiary to joint.prob.multi() below.
    # Inputs: vectors of allele frequencies, f; note, for probability calculations the order of allele
    # frequencies passed to a function matters (see comments below). Naming convention of functions indicate
    # the homozygous/heterozygous status of the pair of genotypes, the number of alleles in common, and the
    # proposed relationship state: Same Individual, Unrelated, Parent Offspring, or Full Sibling.  Probability formulae
    # are taken from Weir et al. (2006).
```

```
# Needed for both biallelic SNPs and MSAT loci with >= 2 alleles at a locus.  Note, due to the biallelic
# nature of SNP markers, probability calculations for pairs of diploid genotypes involving > 2 alleles are not
# needed (i.e. hom.het0, het.het0, and het.het1)
  # Two homozygous genotypes, two shared alleles
  # Arguments: a single allele frequency corresponds to the matching allele
    hom.hom2.SI <- function(f){
      return(f[1]^2)
      } # End joint genotype probability calculation for observing ii/ii given same individual
    hom.hom2.U <- function(f){
      return(f[1]^4)
      } # End joint genotype probability calculation for observing ii/ii given two unrelated individuals
    hom.hom2.PO <- function(f){
      return(f[1]^3)
      } # End joint genotype probability calculation for observing ii/ii given a parent offspring relationship
    hom.hom2.FS <- function(f){
      return(0.25*f[1]^2 + 0.5*f[1]^3 + 0.25 * f[1]^4)
      } # End joint genotype probability calculation for observing ii/ii given two full siblings

  # Two homozygous genotypes, zero shared alleles
  # Arguments: vector of two allele frequencies which correspond to one homozygote and second to the
  # other, order doesn't matter
    hom.hom0.SI <- function(f){
      return(0)
      } # End joint genotype probability calculation for observing ii/jj given same individual
    hom.hom0.U <- function(f){
      return(f[1]^2 * f[2]^2)
      } # End joint genotype probability calculation for observing ii/jj given two unrelated individuals
    hom.hom0.PO <- function(f){
      return(0)
      } # End joint genotype probability calculation for observing ii/jj given a parent offspring relationship
    hom.hom0.FS <- function(f){
      return(0.25*f[1]^2 * f[2]^2)
      } # End joint genotype probability calculation for observing ii/jj given two full siblings

  # One homozygote, one heterozygote, one shared allele
  # Arguments: vector of two allele frequencies, here the first allele frequency must correspond to the
  # homozygous genotype and the second to the novel allele in the heterzygote, e.g. ii/ij => f = c(f_i,f_j).
    hom.het1.SI <- function(f){
      return(0)
      } # End joint genotype probability calculation for observing ii/ij given same individual
    hom.het1.U <- function(f){
      return(2*f[1]^3 * f[2])
      } # End joint genotype probability calculation for observing ii/ij given two unrelated individuals
    hom.het1.PO <- function(f){
      return(f[1]^2 * f[2])
      } # End joint genotype probability calculation for observing ii/ij given a parent offspring relationship
    hom.het1.FS <- function(f){
      return(0.5*f[1]^2 * f[2] + 2*0.25*f[1]^3 * f[2])
      } # End joint genotype probability calculation for observing ii/ij given two full siblings

  # Two heterozygote genotypes, two shared alleles
  # Arguments: a vector of two allele frequencies, the first allele corresponds to one allele in the
  # heterozygote, and the second the other, order doesn't matter
    het.het2.SI <- function(f){
      return(2*f[1] * f[2])
      } # End joint genotype probability calculation for observing ij/ij given same individual
```

```
      het.het2.U <- function(f){
        return(4*f[1]^2 * f[2]^2)
        } # End joint genotype probability calculation for observing ij/ij given two unrelated individuals
      het.het2.PO <- function(f){
        return(f[1] * f[2] * (f[1] + f[2]) )
        } # End joint genotype probability calculation for observing ij/ij given a parent offspring relationship
      het.het2.FS <- function(f){
        return( (2*0.25*f[1] * f[2]) + (0.5*f[1] * f[2] * (f[1] + f[2]) ) + (4*0.25*f[1]^2 * f[2]^2))
        } # End joint genotype probability calculation for observing ij/ij given two full siblings

#--------------------------------------------------------------------------------------------------------------#
# User defined function for intermediary calculations of Pr(k1,k2|R) needed later to caculate the likelihood of a hypothesized
# relationship state between a pair of observed multilocus genotypes.

  # joint.prob.multi() -- This function outputs a dataframe whereby each row defines a proposed pair of diploid genotypes
  # at a locus, and the probability of observing this pair given a true relationship from which the pair of genotypes
  # originated, of Same Individual, Unrelated individuals, Parent Offspring, or Full Sibling.
  # Arguments: f. is a 1-row numeric object (works with 1-row matrix object or 1-row data.frame object with column
  # labels) containing allele frequencies for all alleles at a given locus, and with column
  # names corresponding to unique allele names as single character strings (e.g. lowercase letters).
  # Column naming corresponding to unique allele codes is needed to reference the correct
  #  order of alleles when making probability calculations. Utilizes user defined functions for Pr(k_1,k_2|R)
  # above.

    joint.prob.multi <- function(f.){
      # The total number of unique unordered pairs from a set of n unique elements is: n + choose(n,2) = n + (n!) / (2!(n-2)!)
        G.ord <- combn(x=rep(colnames(f.),2),m=2) # this creates all possible ORDERED diploid genotypes combinations with replacement
        G.unord <- unique(names(table(apply(G.ord,MARGIN=2,FUN=function(x){paste(sort(x),collapse="")})))) # get unique set of unordered pairs
      # A total of, length(G.unord)^2 possible ordered genotype 'dyads' of unique unordered diploid genotypes, with formatting into
      # a dataframe of four columns for four proposed relationship states:
        out <- data.frame(
          k1=rep(G.unord,each=length(G.unord)),
          k2=rep(G.unord,length(G.unord)),
          prob.SI=NA,prob.U=NA,prob.PO=NA,prob.FS=NA)
      # Now set up a series of genotype comparison challenges to come up with appropriate pairwise probability calculation.
      # There are seven total possible allele sharing outcomes to test.
        for(i in 1:nrow(out)){
          # break out each diploid genotype into component alleles for subsequent allele frequency referencing
            g1 <- strsplit(as.character(out$k1[i]), "")[[1]]
            g2 <- strsplit(as.character(out$k2[i]), "")[[1]]
          # hom.hom, 2 shared alleles
            if(length(unique(g1))==1 & length(unique(g2))==1 & length(table(c(g1,g2)))==1){
              out$prob.SI[i] <- hom.hom2.SI(f=f.[1,g1[1]])
              out$prob.U[i] <- hom.hom2.U(f=f.[1,g1[1]])
              out$prob.PO[i] <- hom.hom2.PO(f=f.[1,g1[1]])
              out$prob.FS[i] <- hom.hom2.FS(f=f.[1,g1[1]])
              } else
          # hom.hom, 0 shared alleles
            if(length(unique(g1))==1 & length(unique(g2))==1 & length(table(c(g1,g2)))==2){
              out$prob.SI[i] <- hom.hom0.SI(f=c(f.[1,g1[1]],f.[1,g2[1]]))
              out$prob.U[i] <- hom.hom0.U(f=c(f.[1,g1[1]],f.[1,g2[1]]))
              out$prob.PO[i] <- hom.hom0.PO(f=c(f.[1,g1[1]],f.[1,g2[1]]))
              out$prob.FS[i] <- hom.hom0.FS(f=c(f.[1,g1[1]],f.[1,g2[1]]))
              } else
          # hom.het, 1 shared alleles
            if(length(unique(g1))==1 & length(unique(g2))==2 & length(intersect(g1,g2))==1 |
              length(unique(g1))==2 & length(unique(g2))==1 & length(intersect(g1,g2))==1 ){
```

```
                out$prob.SI[i] <- hom.het1.SI(f=f.[1,names(sort(table(c(g1,g2)),decreasing=T))])
                out$prob.U[i] <- hom.het1.U(f=f.[1,names(sort(table(c(g1,g2)),decreasing=T))])
                out$prob.PO[i] <- hom.het1.PO(f=f.[1,names(sort(table(c(g1,g2)),decreasing=T))])
                out$prob.FS[i] <- hom.het1.FS(f=f.[1,names(sort(table(c(g1,g2)),decreasing=T))])
                } else
            # het.het, 2 shared alleles
              if(length(unique(g1))==2 & length(unique(g2))==2 & length(intersect(g1,g2))==2){
                out$prob.SI[i] <- het.het2.SI(f=f.[1,names(sort(table(c(g1,g2)),decreasing=T))])
                out$prob.U[i] <- het.het2.U(f=f.[1,names(sort(table(c(g1,g2)),decreasing=T))])
                out$prob.PO[i] <- het.het2.PO(f=f.[1,names(sort(table(c(g1,g2)),decreasing=T))])
                out$prob.FS[i] <- het.het2.FS(f=f.[1,names(sort(table(c(g1,g2)),decreasing=T))])
                } # else
            } # end i loop over rows of out matrix
          return(out)
        } # End joint.prob.multi function

        # Examples:
          # Biallelic SNP locus, minor allele frequency = 0.2
            n.allele <- 2
            f.df <- as.data.frame(matrix(nr=1,nc=length(letters[1:n.allele]),c(0.2,0.8)))
            colnames(f.df) <- letters[1:n.allele]
            (joint.prob.multi(f.=f.df))

### Section 2: Example data simulation ##############################################################################

  #--------------------------------------------------------------------------------------------------------------#
  # Simulate diploid "SNP" type genotype data with potential allelic dropout and mistyping genotyping error.

  # SNP.G.simulator() -- this function produces diploid multilocus genotypes from unrelated individuals
  # under a specified set of allele frequencies and genotyping error rates.  A list of objects is
  # returned: the simulated genotypes with a leading column indexing a generic sampling id, and a last column
  # indicating true individual id, followed by all other inputs passed back to the user.
  # Arguments: F.sim.ls = a list object where each element is a 1 row matrix of
  # allele frequencies and with column names indicating allele names (must be single-character strings, e.g.
  # lower case letters or upper case letters) with one row for each locus simulated. Thus, the number of elements
  # in the F.sim.ls sets the number of loci. See example below; ERR.locus =  vector of per-allele
  # generic genotyping error rates for biallelic SNP-type loci; n.unique = total number of unique individuals
  # for which to simualte genotypes; id.start = integer at which unique individual id numbering commences;
  # recap.ix = vector indicating which of 1:n.unique individuals is recaught, which can include repeated
  # captures of individuals (e.g. recap.ix = c(1,1,2,3)).  If left as 'NA', no recaptures are simulated;
  # r.seed = a random number seed. If left as 'NA', then no seed is passed.

  SNP.G.simulator <- function(F.sim.ls,ERR.locus,n.unique,id.start,recap.ix=NA,r.seed=NA){
    # Set seed
      if(is.na(r.seed)==F){set.seed(r.seed)}
    # Translate locus-level error rate to per-allele rates (same for all loci here)
      err <- 1-sqrt(1-ERR.locus)
    # Calculate number recaptures
      if(length(recap.ix)==1 & sum(is.na(recap.ix))==1) {n.recap <- 0} else {n.recap <- length(recap.ix)}
    # Declare indexing varibles
      n.loci <- length(F.sim.ls)
      col.even <- seq(from=2,to=2*n.loci,by=2)
      col.odd <- seq(from=1,to=2*n.loci,by=2)
    # Set up genotype storage object
      G.lat <- data.frame(matrix(nr=n.unique+n.recap,nc=2*n.loci))
    # True genotypes for n.unique individuals
      for(j in 1:n.loci){
```

```
        G.lat[1:n.unique,col.odd[j]] <- sample(colnames(F.sim.ls[[j]]),size=n.unique,prob=F.sim.ls[[j]],replace=T)
        G.lat[1:n.unique,col.even[j]] <- sample(colnames(F.sim.ls[[j]]),size=n.unique,prob=F.sim.ls[[j]],replace=T)
        } # end j loop over loci
    # Simulate recaptured samples and populate sample and true identifications
      if(n.recap>0) {G.lat[(n.unique+1):(n.unique+n.recap),] <- G.lat[recap.ix,]}
    # Generate observed genotypes, following generic biallelic SNP genotyping error model (see Supplementary Text 1).
      G.obs <- G.lat
      for(j in 1:n.loci){
        # first allele copy for locus j, across all rows of G matrix
          temp.err <- rbinom(n=nrow(G.lat),size=1,prob=err[j])
          G.obs[temp.err==1,col.odd[j]] <- sapply(G.obs[temp.err==1,col.odd[j]],FUN=function(x){
            sample(colnames(F.sim.ls[[j]])[colnames(F.sim.ls[[j]])!=x],size=1,prob=F.sim.ls[[j]][colnames(F.sim.ls[[j]])!=x],
            replace=T)})
        # second allele copy for locus j, across all rows of G matrix
          temp.err <- rbinom(n=nrow(G.lat),size=1,prob=err[j])
          G.obs[temp.err==1,col.even[j]] <- sapply(G.obs[temp.err==1,col.even[j]],FUN=function(x){
            sample(colnames(F.sim.ls[[j]])[colnames(F.sim.ls[[j]])!=x],size=1,prob=F.sim.ls[[j]][colnames(F.sim.ls[[j]])!=x],
            replace=T)})
        } # end j loop over loci for genotyping error
    # Add in columns for sample vs. true identification
      if(n.recap>0){G.obs <- cbind(id.start:(id.start-1 + n.unique + n.recap),G.obs,
        c(id.start:(id.start-1+n.unique),(id.start:(id.start-1 + n.unique))[recap.ix]))} else
        {G.obs <- cbind(id.start:(id.start-1 + n.unique),G.obs,id.start:(id.start-1 + n.unique))}
      colnames(G.obs)[c(1,ncol(G.obs))] <- c("Sample.ID","True.ID")
      return(structure(list(G.obs,F.sim.ls,ERR.locus,n.unique,recap.ix,r.seed),
        .Names=c("G.obs","F.sim.ls","ERR.locus","n.unique","recap.ix","r.seed")))
      } # end function SNP.G.simulator()

  # Examples:
  # Generate an example data set for stage-1 clustering with which to identify within-sampling occasion recaptures.
  # 64 biallelic "SNP" loci each 0.3 minor allele frequency, a 2% generic per locus genotyping error rate.
  # Generate multilocus genotypes for 15 unique unrelated individuals, a double recapture of individual 1,
  # and single recaptures of individuals 2-4.
  # Simulation parameters:
    num.loci=64; maf=0.3; allele.names=letters[1:2]
    F.ls <- lapply(1:num.loci,FUN=function(j){
                x <- t(as.matrix(c(maf,1-maf)));
                colnames(x) <- allele.names;
                return(x)})
    Ss1.sim <- SNP.G.simulator(F.sim.ls=F.ls, ERR.locus = rep(0.02,num.loci), n.unique = 15,
      id.start=100,recap.ix = c(1,1,2,3,4),r.seed = 1)

  # Generate a pair of genotype data sets for testing of stage-2 sample clustering to identify between-sampling occasion
  # recaptures.  Set 1, as above but made up of 20 unique individuals.  Set 2 made up of 5 recaptures from Set 1, and
  # 15 unique unrelated individuals.
    Ss2_1.sim <- SNP.G.simulator(F.sim.ls=F.ls, ERR.locus = rep(0.02,num.loci), n.unique = 20,
      id.start=100,recap.ix = NA,r.seed = 2)
    Ss2_2.sim <- SNP.G.simulator(F.sim.ls=F.ls, ERR.locus = rep(0.02,num.loci), n.unique = 15,
      id.start=200,recap.ix = NA,r.seed = 3)
    Ss2_2.sim$G.obs <- rbind(Ss2_2.sim$G.obs,Ss2_1.sim$G.obs[1:5,]) # create recaptures in sampling occasion 2
    # repopulate a generic sample ID number for occasion 2
    Ss2_2.sim$G.obs$Sample.ID <- Ss2_2.sim$G.obs$Sample.ID[1]:(Ss2_2.sim$G.obs$Sample.ID[1] + nrow(Ss2_2.sim$G.obs)-1)

### Section 3: Stage-one clustering to identify repeated captures within a single sampling occasion ###########################
#-------------------------------------------------------------------------------------------------------------#
# Inputs
  # Get genotype data from a single capture occasion.
```

```
  # NOTE: Input genotype data need have in column 1 a sample ID, followed by a column for
  # each allele copy for each diploid locus.  Allele calls need be single character strings.
  # See example output from SNP.G.simulator() for input genotype formatting examples.
    S.input <- Ss1.sim$G.obs[,1:(ncol(Ss1.sim$G.obs)-1)] # Don't need the true individual ID column
  # Get allele frequency matrix.  Format: a list object where each element is a 1 row matrix of allele
  # frequencies and with  column names indicating allele nameswith one row for each locus simulated. Thus,
  # the number of elements in the F.sim.ls sets the number of loci.
    F.m <- Ss1.sim$F.sim.ls # F.sim.m
  # Declare vectors of locus-level error rates
    err.l <- Ss1.sim$ERR.locus

#--------------------------------------------------------------------------------------------------------------#
# Initialize intermediate quantities and storage objects
  # Get number of alleles at each locus
    n.allele <- sapply(F.m,FUN=function(x){length(x)})
  # Get number of loci
    n.loci <- length(F.m)
  # Convert locus-level error rates to per-allele rates
    err <- 1-sqrt(1-err.l)
  # Given the input allele frequencies, define joint probabilities given different relationship states,
  # using the joint.prob.multi() user defined function. Can take time to generate if many alleles at loci.
  # The idea is to calculate this once, and then reference relevant genotype pairs during the clustering
  # loop later.
    joint.prob.ls <- list()
    for(L in 1:length(F.m)){
      joint.prob.ls[[L]] <- joint.prob.multi(f.=F.m[[L]])
      }
  # Column indexing vector--be careful here, this relies on specific format of input genotypes (see above).
    col.even <- seq(from=2,to=ncol(S.input)-1,by=2)
    col.odd <- seq(from=3,to=ncol(S.input),by=2)
  # Initialize storage variables if possible outside of loops below, helper functions
    n.prob.ls <- 9 # 9 possible pairs of diploid genotypes with biallelic markers
    # Storage vector, defined relevant to number of alleles at locus j
      Pg1_k1 <- rep(1,n.prob.ls)
      Pg2_k2 <- Pg1_k1
    # Storage vectors for discrepancies between pairs of genotypes
      dg1_k1 <- rep(0,n.prob.ls)
      dg2_k2 <- dg1_k1
  # Helper function
    `%notin%`<- Negate(`%in%`)
  # Storage matrix for L(observed genotypes in pair of sets | relationship) at each locus, j.
  # A matrix of dimension rows = # loci, cols = 4 (in order, SI, U, PO, FS)
    LRG1G2j <- matrix(nr=n.loci,nc=4,1)
    colnames(LRG1G2j) <- c("SI","U","PO","FS") # assign colnames to object for record keeping/debugging
  # Storage for Likelihood product across all (assumed independent) loci
    LRG1G2 <- 1:4 # L| SI, U, PO, FS

#--------------------------------------------------------------------------------------------------------------#
# Stage-one clustering in action. This code contains several error traps; see code comments below.
  # Step 1: Intialize the clustering algorithm with all samples as singleton sets, a list object with sample IDs.
    S <- list()
    for(z in 1:nrow(S.input)){S[[z]] <- S.input[z,1]} # sample ID taken from first column
    S.old <- S # temporary copy
  # Steps 2-3: Pairwise comparisons for match calls, repeating until set membership stops changing
     repeat{
      S.old <- S
      for(i in 1:length(S)){
```

```
# Likelihood ratio and set membership sample ID labels placeholders; need list objects here s.t.
# a given element can hold more than one multilocus genotype if need be.
  temp.rat.sc <- list()
  temp.rat.mr <- list()
# Indexing variable place holder
  ix1 <- 0
# Exit trap, don't make a match comparison if S[[i]] set is NA, i.e. G1 is empty set
  if(sum(is.na(S[[i]]))>0) {next}
# Indexing construct for i and k>i results in all unique pairwise comparisons across sets
  for(k in (1:length(S))[(1:length(S))>i]){
    # Get the pair of sets of genotypes to compare
      G1 <- S.input[S.input[,1]%in%S[[i]],]
      G2 <- S.input[S.input[,1]%in%S[[k]],]
    # Exit trap, if G2 is empty set proceed to next k
      if(nrow(G2)==0) {next}
    # Determine which are common positive PCR loci, i.e. the set of loci for which both samples
    # have any genotype call. Note, the below code still works when a given G1 or G2 set has multiple
    # elements.
      pos.loci.ix <- col.even[(nrow(G1)-colSums(is.na(G1[col.even])))>0 &
        (nrow(G2)-colSums(is.na(G2[col.even])))>0]/2
    # Update indexing variable
      ix1 <- ix1+1
    # Exit trap, if pos.loci.ix is zero, implying no common loci with genotypes which can happen with real-world
    # data with failed PCR outcomes across some loci.  One could impose a common-loci minimum threshold here
    # as well. Note, exit in this case results in retaining separation of the two compared sample sets, implying
    # they derive from separate individuals.
      if(length(pos.loci.ix)==0) {
        # store index information for compared sets, and evidence ratio
        temp.rat.sc[[ix1]] <- G2[,1]
        temp.rat.mr[[ix1]] <- 0 # force to zero
        next
        } # end if
    # Make calculations for likelihood across loci, Lj(R_(1,2)), for each locus
    for(j in 1:n.loci){
      # First, determine whether there is missing genotype info among the two compared sets at this locus,
      # using pos.loci.ix from above.  If yes, pass a 1.0 value which will not affect likelihood
      # multiplication subsequently (i.e. Like * 1 = Like).
        if(j %notin% pos.loci.ix){
          LRG1G2j[j,1:4] <- rep(1,4)
          next} # end if; 'next' goes to next locus
      # Reference the set of possible pairs of genotypes at a given locus and associated joint probabilities
      # calculated with joint.prob.multi() outside the clustering algorithm, Pr(kj1,kj2|R),
        temp.prob <- joint.prob.ls[[j]]
      # Calculate the probability of observed genotypes given proposed latent genotypes, Pr(gj|kj).
      # This code accomodates multiple genotypes within a set, e.g. if after an
      # iteration of the clustering algorithm, it is found that two samples from a single individual are
      # grouped into one set.
        # Reset storage vector for probabilities of pairs of genotypes to 1.0
          Pg1_k1[] <- rep(1,n.prob.ls)
          Pg2_k2[] <- rep(1,n.prob.ls)
        # Reset storage vectors for discrepancies between pairs of genotypes to zero
          dg1_k1[] <- rep(0,n.prob.ls)
          dg2_k2[] <- rep(0,n.prob.ls)
        for(ii in 1:nrow(G1)){
          # First, determine whether there is a missing locus; if yes, then exit
            if(sum(is.na(G1[ii,(col.even[j]:col.odd[j])]))>0){next}
          # Else compute number of discrepant alleles between all g1 in G1 and k1's.  This code
```

```
          # sorts alleles so that all heterozygotes have the same form, e.g. "ba" sorted to "ab".
          # This is  necessary to correctly reference latent genotypes from the joint.prob.ls object.
            dg1_k1[1:n.prob.ls] <- dg1_k1[1:n.prob.ls] +
              adist(paste0(sort(G1[ii,(col.odd[j]:col.even[j])]),collapse=""),temp.prob$k1)
          }
        for(ii in 1:nrow(G2)){
            if(sum(is.na(G2[ii,(col.even[j]:col.odd[j])]))>0){next}
            dg2_k2[1:n.prob.ls] <- dg2_k2[1:n.prob.ls] +
              adist(paste0(sort(G2[ii,(col.odd[j]:col.even[j])]),collapse=""),temp.prob$k2)
          }
        # With biallelic SNP error model, calculate Pr(gj|kj)
          Pg1_k1[1:9] <- sapply(dg1_k1,FUN=function(x){P.error.snp(gamma_j=err[j],n.a=2*nrow(G1),d_gk=x)})
          Pg2_k2[1:9] <- sapply(dg2_k2,FUN=function(x){P.error.snp(gamma_j=err[j],n.a=2*nrow(G2),d_gk=x)})
      # Crossproducts to sum over all combinations of Pr(k1k2|R) * Pr(g1|k1) * Pr(g2|k2) at this locus
        LRG1G2j[j,1] <- as.numeric(temp.prob$prob.SI) %*% (Pg1_k1*Pg2_k2)
        LRG1G2j[j,2] <- as.numeric(temp.prob$prob.U) %*% (Pg1_k1*Pg2_k2)
        LRG1G2j[j,3] <- as.numeric(temp.prob$prob.PO) %*% (Pg1_k1*Pg2_k2)
        LRG1G2j[j,4] <- as.numeric(temp.prob$prob.FS) %*% (Pg1_k1*Pg2_k2)
      } # end j loop over loci
    # Now product across loci, L(R_(1,2))
      LRG1G2 <- apply(LRG1G2j,MARGIN=2,FUN=prod)
    # Store index information for compared sets, and likelihood ratios of L(R_(1,2)=SI)/L(R_(1,2)=other).
    # See Wang(2004,2006,2016), Kalinowski et al. (2006), and Supplement 1 to this article for details
    # on the sample matching likelihood ratio model.
      temp.rat.sc[[ix1]] <- G2[,1]
      temp.rat.mr[[ix1]] <- LRG1G2[1]/max(LRG1G2[2:4],na.rm=T)
      } # end k loop over all samples > i (pairwise combos)
  # Update set membership by combining G2 into G1 if max likelihood ratio > 1.0
    if(length(temp.rat.mr)>0){
      # As written here, temp.Sample.Code can be multiple different sets if more than one
      # set produces a max evidence ratio
        temp.rat.mr.v <- unlist(temp.rat.mr)
        temp.Sample.Code <-
          unlist(temp.rat.sc[temp.rat.mr.v==max(temp.rat.mr.v,na.rm=T) & temp.rat.mr.v>1.0])
          } else {temp.Sample.Code <- NULL}
        S[[i]] <- c(S[[i]],temp.Sample.Code)
    # Now  remove the sample codes from the singleton sets such that it isn't considered for
    # allocation again later
      if(length(temp.Sample.Code)>0){
        S[unlist( lapply(S,FUN=function(x){sum(x%in%temp.Sample.Code)==length(x)}) )] <- NA
        } # end if
    } # end i loop
  S <- lapply(S, function(x) x[!is.na(x)]) # Cleaning up
  S <- S[lapply(S,length)>0] # Cleaning up
  if(identical(S,S.old)==TRUE){break}
} # End repeat when no new updating, step 4.

# Convert the final S list to a matrix object to examine clustering
  S.m <- matrix(nr=length(S),nc=max(unlist(lapply(S,FUN=length))),NA )
  for(r in 1:nrow(S.m)){
    S.m[r,1:length(S[[r]])] <- S[[r]]
    } # end r loop
  # print
    S.m # Unique individuals along rows, any corresponding recaptures' Sample IDs in column 2+
    Ss1.sim$G.obs[,c("Sample.ID","True.ID")] # Cross reference Sample IDs to verify recapture identity

### Section 4: Stage two clustering to identify recaptures across sampling occasions #################################
```

```
#-------------------------------------------------------------------------------------------------------------#
# Inputs
  # Get genotype data from a pair of capture occasions.
  # NOTE: Input genotype data need have in column 1 a sample ID, followed by a column for
  # each allele copy for each diploid locus.  Allele calls need be single character strings.
  # See example output from SNP.G.simulator() for input genotype formatting examples.
    S1.input <- Ss2_1.sim$G.obs[,1:(ncol(Ss2_1.sim$G.obs)-1)] # Don't need the true individual ID column
    S2.input <- Ss2_2.sim$G.obs[,1:(ncol(Ss2_2.sim$G.obs)-1)] # Don't need the true individual ID column
  # Get allele frequency matrix.  Format: a list object where each element is a 1 row matrix of allele
  # frequencies and with  column names indicating allele nameswith one row for each locus simulated. Thus,
  # the number of elements in the F.sim.ls sets the number of loci.
    F.m <- Ss1.sim$F.sim.ls # F.sim.m
  # Declare vectors of locus-level error rates
    err.l <- Ss1.sim$ERR.locus

#-------------------------------------------------------------------------------------------------------------#
# Initialize intermediate quantities and storage objects
  # Get number of alleles at each locus
    n.allele <- sapply(F.m,FUN=function(x){length(x)})
  # Get number of loci
    n.loci <- length(F.m)
  # Convert locus-level error rates to per-allele rates
    err <- 1-sqrt(1-err.l)
  # Given the input allele frequencies, define joint probabilities given different relationship states,
  # using the joint.prob.multi() user defined function. Can take time to generate if many alleles at loci.
  # The idea is to calculate this once, and then reference relevant genotype pairs during the clustering
  # loop later.
    joint.prob.ls <- list()
    for(L in 1:length(F.m)){
      joint.prob.ls[[L]] <- joint.prob.multi(f.=F.m[[L]])
      }
  # Column indexing vector--be careful here, this relies on specific format of input genotypes (see above).
    col.even <- seq(from=2,to=ncol(S.input)-1,by=2)
    col.odd <- seq(from=3,to=ncol(S.input),by=2)
  # Initialize storage variables if possible outside of loops below, helper functions
    n.prob.ls <- 9 # 9 possible pairs of diploid genotypes with biallelic markers
    # Storage vector, defined relevant to number of alleles at locus j
      Pg1_k1 <- rep(1,n.prob.ls)
      Pg2_k2 <- Pg1_k1
    # Storage vectors for discrepancies between pairs of genotypes
      dg1_k1 <- rep(0,n.prob.ls)
      dg2_k2 <- dg1_k1
  # Helper function
    `%notin%`<- Negate(`%in%`)
  # Storage matrix for L(observed genotypes in pair of sets | relationship) at each locus, j.
  # A matrix of dimension rows = # loci, cols = 4 (in order, SI, U, PO, FS)
    LRG1G2j <- matrix(nr=n.loci,nc=4,1)
    colnames(LRG1G2j) <- c("SI","U","PO","FS") # assign colnames to object for record keeping/debugging
  # Storage for Likelihood product across all (assumed independent) loci
    LRG1G2 <- 1:4 # L| SI, U, PO, FSS

#-------------------------------------------------------------------------------------------------------------#
# Stage-two clustering in action. This code contains several error traps; see code comments below.
  # Intialize the clustering algorithm with all samples as singleton sets, list objects with sample IDs.
    S1 <- list()
    S2 <- list()
    for(z in 1:nrow(S1.input)){S1[[z]] <- S1.input[z,1]} # sample ID is in first column
```

```
  for(z in 1:nrow(S2.input)){S2[[z]] <- S2.input[z,1]}
# Steps 1-2: Pairwise comparisons for match calls
    for(i in 1:length(S1)){
      # Likelihood ratio and set membership sample ID labels placeholders; need list objects here s.t.
      # a given element can hold more than one multilocus genotype if need be.
        temp.rat.sc <- list()
        temp.rat.mr <- list()
      # Indexing variable place holder
        ix1 <- 0
      # Exit trap, don't make a match comparison if S1[[i]] set is NA, i.e. G1 is empty set
        if(sum(is.na(S1[[i]]))>0) {next}
      # Indexing across sampling occaions, compare each ith sample in S1 to all samples in S2 for recaptures
        for(k in 1:length(S2)){
          # Get the pair of sets of genotypes to compare
            G1 <- S1.input[S1.input[,1]%in%S1[[i]],]
            G2 <- S2.input[S2.input[,1]%in%S2[[k]],]
          # Exit trap, if G2 is empty set proceed to next k
            if(nrow(G2)==0) {next}
          # Determine which are common positive PCR loci, i.e. the set of loci for which both samples
          # have any genotype call. Note,the below code still works when a given G1 or G2 set has multiple
          # elements
            pos.loci.ix <- col.even[(nrow(G1)-colSums(is.na(G1[col.even])))>0 &
              (nrow(G2)-colSums(is.na(G2[col.even])))>0]/2
          # Update indexing variable
            ix1 <- ix1+1
          # Exit trap, if pos.loci.ix is zero, implying no common loci with genotypes which can happen with real-world
          # data with failed PCR outcomes across some loci.  One could impose a common-loci minimum threshold here
          # as well. Note, exit in this case results in retaining separation of the two compared sample sets, implying
          # they derive from separate individuals.
            if(length(pos.loci.ix)==0) {
              # store index information for compared sets, and evidence ratio
              temp.rat.sc[[ix1]] <- G2[,1]
              temp.rat.mr[[ix1]] <- 0 # force to zero
              next
              } # end if
          # Make calculations for likelihood across loci, Lj(R_(1,2)), for each locus
          for(j in 1:n.loci){
            # First, determine whether there is missing genotype info among the two compared sets at this locus,
            # using pos.loci.ix from above.  If yes, pass a 1.0 value which will not affect likelihood
            # multiplication subsequently (i.e. Like * 1 = Like).
              if(j %notin% pos.loci.ix){
                LRG1G2j[j,1:4] <- rep(1,4)
                next} # end if; 'next' goes to next locus
            # Reference the set of possible pairs of genotypes at a given locus and associated joint probabilities
            # calculated with joint.prob.multi() outside the clustering algorithm, Pr(kj1,kj2|R),
              temp.prob <- joint.prob.ls[[j]]
            # Calculate the probability of observed genotypes given proposed latent genotypes, Pr(gj|kj).
            # This code accomodates multiple genotypes within a set, e.g. if after an
            # iteration of the clustering algorithm, it is found that two samples from a single individual are
            # grouped into one set.
              # Reset storage vector for probabilities of pairs of genotypes to 1.0
                Pg1_k1[] <- rep(1,n.prob.ls)
                Pg2_k2[] <- rep(1,n.prob.ls)
              # Reset storage vectors for discrepancies between pairs of genotypes to zero
                dg1_k1[] <- rep(0,n.prob.ls)
                dg2_k2[] <- rep(0,n.prob.ls)
              for(ii in 1:nrow(G1)){
```

```
          # First, determine whether there is a missing locus; if yes, then exit
            if(sum(is.na(G1[ii,(col.even[j]:col.odd[j])]))>0){next}
          # Else compute number of discrepant alleles between all g1 in G1 and k1's.  This code
          # sorts alleles so that all heterozygotes have the same form, e.g. "ba" sorted to "ab".
          # This is  necessary to correctly reference latent genotypes from the joint.prob.ls object.
            dg1_k1[1:n.prob.ls] <- dg1_k1[1:n.prob.ls] +
              adist(paste0(sort(G1[ii,(col.odd[j]:col.even[j])]),collapse=""),temp.prob$k1)
          }
        for(ii in 1:nrow(G2)){
            if(sum(is.na(G2[ii,(col.even[j]:col.odd[j])]))>0){next}
            dg2_k2[1:n.prob.ls] <- dg2_k2[1:n.prob.ls] +
              adist(paste0(sort(G2[ii,(col.odd[j]:col.even[j])]),collapse=""),temp.prob$k2)
          }
        # With biallelic SNP error model, calculate Pr(gj|kj)
          Pg1_k1[1:9] <- sapply(dg1_k1,FUN=function(x){P.error.snp(gamma_j=err[j],n.a=2*nrow(G1),d_gk=x)})
          Pg2_k2[1:9] <- sapply(dg2_k2,FUN=function(x){P.error.snp(gamma_j=err[j],n.a=2*nrow(G2),d_gk=x)})
      # Crossproducts to sum over all combinations of Pr(k1k2|R) * Pr(g1|k1) * Pr(g2|k2) at this locus
        LRG1G2j[j,1] <- as.numeric(temp.prob$prob.SI) %*% (Pg1_k1*Pg2_k2)
        LRG1G2j[j,2] <- as.numeric(temp.prob$prob.U) %*% (Pg1_k1*Pg2_k2)
        LRG1G2j[j,3] <- as.numeric(temp.prob$prob.PO) %*% (Pg1_k1*Pg2_k2)
        LRG1G2j[j,4] <- as.numeric(temp.prob$prob.FS) %*% (Pg1_k1*Pg2_k2)
      } # end j loop over loci
  # Now product across loci, L(R_(1,2))
    LRG1G2 <- apply(LRG1G2j,MARGIN=2,FUN=prod)
  # Store index information for compared sets, and likelihood ratios of L(R_(1,2)=SI)/L(R_(1,2)=other).
  # See Wang(2004,2006,2016), Kalinowski et al. (2006), and Supplement 1 to this article for details
  # on the sample matching likelihood ratio model.
    temp.rat.sc[[ix1]] <- G2[,1]
    temp.rat.mr[[ix1]] <- LRG1G2[1]/max(LRG1G2[2:4],na.rm=T)
    } # end k loop over all samples > i (pairwise combos)
# Update set membership by combining G2 into G1 if max likelihood ratio > 1.0
  if(length(temp.rat.mr)>0){
    # As written here, temp.Sample.Code can be multiple different sets if more than one
    # set produces a max evidence ratio
      temp.rat.mr.v <- unlist(temp.rat.mr)
      temp.Sample.Code <-
        unlist(temp.rat.sc[temp.rat.mr.v==max(temp.rat.mr.v,na.rm=T) & temp.rat.mr.v>1.0])
        } else {temp.Sample.Code <- NULL}
      S1[[i]] <- c(S1[[i]],temp.Sample.Code)
  # Now  remove the sample codes from the singleton sets in S2 such that it isn't considered for
  # allocation again later
    if(length(temp.Sample.Code)>0){
      S2[unlist( lapply(S2,FUN=function(x){sum(x%in%temp.Sample.Code)==length(x)}) )] <- NA
      } # end if
  } # end i loop
S2 <- lapply(S2, function(x) x[!is.na(x)]) # Cleaning up
S2 <- S2[lapply(S2,length)>0] # Cleaning up

# Convert the final S1 list to a matrix object to examine clustering.  Those rows (sets) with > 2 members
# indicate the sample IDs captured in both occasion one and occasion
S.m <- matrix(nr=length(S1),nc=max(unlist(lapply(S1,FUN=length))),NA )
for(r in 1:nrow(S.m)){
  S.m[r,1:length(S1[[r]])] <- S1[[r]]
  } # end r loop
# print
  S.m # Sample IDs from Set 1 in column 1 and any corresponding recaptures' Sample IDs in column 2
  Ss2_2.sim$G.obs[,c("Sample.ID","True.ID")] # Cross reference Sample IDs to verify recapture identity
```

```
### Section 5: References ###################################################################################################
  # Kalinowski ST, Taper ML, Creel S. 2006 Using DNA from non-invasive
    # samples to identify individuals and census populations: an evidential
    # approach tolerant of genotpying errors. Conserv. Genet. 7, 319-329.
  # Wang J. 2004 Sibship reconstruction from genetic data with typing errors.
    # Genetics 166, 1963-1979.
  # Wang J. 2006 Informativeness of genetic markers for pairwise relationship
    # and relatedness inference. Theor. Popul. Biol. 70, 300-321.
  # Wang J. 2016 Individual identification from genetic marker data: developments
    # and accuracy comparisons of methods. Mol. Ecol. Resour. 16, 163-175.
  # Weir B, Anderson AD, Hepler AB. 2006 Genetic relatedness analysis: modern
    # data and new challenges. Nat. Rev. Genet. 7, 771-780.
```

Supplement 4: Case study genetic marker characteristics

Table S4.1 Allele frequencies and genotyping error rates for a sample of Pacific Walrus case study data.

Locus	Minor allele frequency	Total repeated amplifications	Genotyping error rate
R022436_F	0.493	209	0.002
Oro_29_19932	0.487	133	0.004
Oro_26_19246	0.482	151	0.003
R085783_F	0.481	210	0.000
R071991_F	0.479	214	0.000
R095477_R	0.478	211	0.000
R057919_F	0.474	211	0.002
R041755_F	0.469	213	0.000
R063501_R	0.464	209	0.002
Oro_244_6146	0.464	213	0.000
R054658_F	0.460	209	0.007
R069960_F	0.460	213	0.000
Oro_209_4700	0.455	214	0.000
R003929_R	0.453	212	0.000
R110616_R	0.451	212	0.000
Oro_297_12481	0.449	134	0.004
Oro_17_7095	0.449	144	0.010
R074886_R	0.446	213	0.000
R030422_F	0.445	132	0.000
Oro_59_28272	0.445	150	0.007
R044524_F	0.441	147	0.007
Oro_91_81195	0.441	134	0.004
R036451_F	0.439	150	0.020
R076553_F	0.438	214	0.000
R003503_F	0.434	209	0.000
Oro_62_42699	0.431	142	0.035
R015125_F	0.428	213	0.000
R112147_F	0.424	134	0.004
R035683_R	0.424	134	0.007
R050977_R	0.423	212	0.002
R082930_F	0.420	134	0.000
R009365_F	0.418	213	0.000
Oro_200_64743	0.417	134	0.004
R000414_R	0.409	152	0.020
Oro_260_4294	0.408	134	0.004
R045452_R	0.400	212	0.005
R113465_F	0.398	214	0.000
R093661_R	0.395	212	0.000
R016631_R	0.389	211	0.002
R003141_F	0.385	136	0.022
R010659_R	0.378	132	0.004
R007106_F	0.376	129	0.051
R109577_F	0.373	152	0.007
R127210_F	0.369	134	0.004
R024627_R	0.368	212	0.000
Oro_39_28704	0.366	152	0.010

R072907_F	0.363	134	0.000
R053490_F	0.359	134	0.004
R026226_F	0.355	142	0.004
R097533_R	0.353	209	0.000
Oro_82_103979	0.352	137	0.036
R006728_R	0.352	134	0.004
R026061_R	0.352	134	0.000
R022808_R	0.346	209	0.000
R098501_F	0.340	209	0.000
R026855_F	0.333	213	0.000
R013534_F	0.329	213	0.000
R119762_R	0.329	134	0.004
R032094_F	0.325	213	0.000
R072666_R	0.319	152	0.007
R008033_F	0.313	212	0.000
Oro_133_7746	0.243	212	0.000
R083252_FXY	0.124	150	0.003
Oro_18_45046[d]	0.000	65	0.000

[a]Data are from the U.S. Fish and Wildlife Service and represent pilot data (*n*=3824) for two sampling occasions, one in summer 2013 and one in summer 2014 in the Chuchki and Bering Sea, Alaska, U.S.A. Samples were genotyped using Applied Biosystems Custom TaqMan® SNP Genotyping Assays on the QuantStudio™ 12K Flex. Any use of trade, firm, or product names is for descriptive purposes only and does not imply endorsement by the U.S. Government.
[b]Total number of repeated amplifications of genotypes used to assess per-locus genotyping error rates.
[c]Per-locus error rate.
[d]This locus exhibited a single allele and was subsequently purged from matching analysis.

Table S4.2 Allele frequencies and genotyping error rates for a sample of Fisher case study data.

Locus	Number of alleles	Allele frequencies	Total 3x replicates	Allelic d
Lut604	6	{0.3144,0.2776,0.1839,0.087,0.0786,0.0585}	280	0.
Ma1	8	{0.3428,0.3294,0.2592,0.0435,0.0151,0.005,0.0033,0.0017}	275	0.
MP0055	5	{0.5567,0.3233,0.0883,0.0267,0.005}	281	0.
MP0182	6	{0.43,0.2667,0.245,0.0467,0.01,0.0017}	278	0.
Mvis072	6	{0.5035,0.2944,0.1063,0.0697,0.0192,0.007}	258	0.
RIO20	6	{0.3435,0.2058,0.2041,0.1854,0.0357,0.0255}	263	0.
Ggu101	6	{0.6132,0.1807,0.1622,0.0304,0.0118,0.0017}	277	0.
MP0100	5	{0.7299,0.1711,0.0906,0.0067,0.0017}	279	0.
MP0084	7	{0.3688,0.299,0.1993,0.1213,0.005,0.005,0.0017}	280	0.

[a]Data are from the New York Cooperative Fish and Wildlife Research Unit Cornell University, and represent data (*n* = 200) for two week-long sampling occasions at 608 hair snare traps during the winter of 2014 in the state of New York, U.S.A. A total of 302 samples retained in the final study at large were used to calculate allele frequencies; a subset of 281 samples were used to assess genotyping error rates.
[b]Total number of samples repeat amplified three times and used to assess per-locus genotyping error rates.
[c]Per-locus error rate, assessed at the level of a replicate.

Supplement 5: Detailed base case simulation results

Table S5.1 SNP base case simulation results[a].

Relationship state	Number of loci	MAF	Error rate	Match call rate
FS	32	0.2	0.00	0.0001
FS	48	0.2	0.00	0.0000
FS	64	0.2	0.00	0.0000
FS	80	0.2	0.00	0.0000
FS	96	0.2	0.00	0.0000
FS	128	0.2	0.00	0.0000
FS	32	0.2	0.01	0.0038
FS	48	0.2	0.01	0.0006
FS	64	0.2	0.01	0.0000
FS	80	0.2	0.01	0.0000
FS	96	0.2	0.01	0.0000
FS	128	0.2	0.01	0.0000
FS	32	0.2	0.02	0.0061
FS	48	0.2	0.02	0.0007
FS	64	0.2	0.02	0.0003
FS	80	0.2	0.02	0.0001
FS	96	0.2	0.02	0.0000
FS	128	0.2	0.02	0.0000
FS	32	0.2	0.05	0.0167
FS	48	0.2	0.05	0.0043
FS	64	0.2	0.05	0.0011
FS	80	0.2	0.05	0.0003
FS	96	0.2	0.05	0.0001
FS	128	0.2	0.05	0.0000
FS	32	0.2	0.10	0.0334
FS	48	0.2	0.10	0.0136
FS	64	0.2	0.10	0.0037
FS	80	0.2	0.10	0.0016
FS	96	0.2	0.10	0.0010
FS	128	0.2	0.10	0.0001
FS	32	0.2	0.25	0.1254
FS	48	0.2	0.25	0.0791
FS	64	0.2	0.25	0.0523
FS	80	0.2	0.25	0.0335
FS	96	0.2	0.25	0.0221
FS	128	0.2	0.25	0.0104
FS	32	0.3	0.00	0.0000
FS	48	0.3	0.00	0.0000
FS	64	0.3	0.00	0.0000
FS	80	0.3	0.00	0.0000
FS	96	0.3	0.00	0.0000
FS	128	0.3	0.00	0.0000
FS	32	0.3	0.01	0.0009
FS	48	0.3	0.01	0.0001
FS	64	0.3	0.01	0.0000
FS	80	0.3	0.01	0.0000
FS	96	0.3	0.01	0.0000
FS	128	0.3	0.01	0.0000
FS	32	0.3	0.02	0.0021
FS	48	0.3	0.02	0.0002

FS	64	0.3	0.02	0.0000
FS	80	0.3	0.02	0.0000
FS	96	0.3	0.02	0.0000
FS	128	0.3	0.02	0.0000
FS	32	0.3	0.05	0.0079
FS	48	0.3	0.05	0.0012
FS	64	0.3	0.05	0.0001
FS	80	0.3	0.05	0.0001
FS	96	0.3	0.05	0.0000
FS	128	0.3	0.05	0.0000
FS	32	0.3	0.10	0.0224
FS	48	0.3	0.10	0.0064
FS	64	0.3	0.10	0.0025
FS	80	0.3	0.10	0.0007
FS	96	0.3	0.10	0.0003
FS	128	0.3	0.10	0.0000
FS	32	0.3	0.25	0.1189
FS	48	0.3	0.25	0.0753
FS	64	0.3	0.25	0.0477
FS	80	0.3	0.25	0.0311
FS	96	0.3	0.25	0.0193
FS	128	0.3	0.25	0.0084
FS	32	0.4	0.00	0.0000
FS	48	0.4	0.00	0.0000
FS	64	0.4	0.00	0.0000
FS	80	0.4	0.00	0.0000
FS	96	0.4	0.00	0.0000
FS	128	0.4	0.00	0.0000
FS	32	0.4	0.01	0.0006
FS	48	0.4	0.01	0.0000
FS	64	0.4	0.01	0.0000
FS	80	0.4	0.01	0.0000
FS	96	0.4	0.01	0.0000
FS	128	0.4	0.01	0.0000
FS	32	0.4	0.02	0.0013
FS	48	0.4	0.02	0.0001
FS	64	0.4	0.02	0.0001
FS	80	0.4	0.02	0.0000
FS	96	0.4	0.02	0.0000
FS	128	0.4	0.02	0.0000
FS	32	0.4	0.05	0.0050
FS	48	0.4	0.05	0.0010
FS	64	0.4	0.05	0.0003
FS	80	0.4	0.05	0.0001
FS	96	0.4	0.05	0.0001
FS	128	0.4	0.05	0.0000
FS	32	0.4	0.10	0.0185
FS	48	0.4	0.10	0.0057
FS	64	0.4	0.10	0.0016
FS	80	0.4	0.10	0.0004
FS	96	0.4	0.10	0.0001
FS	128	0.4	0.10	0.0000
FS	32	0.4	0.25	0.1158
FS	48	0.4	0.25	0.0790
FS	64	0.4	0.25	0.0480
FS	80	0.4	0.25	0.0294

FS	96	0.4	0.25	0.0204
FS	128	0.4	0.25	0.0101
SI	32	0.2	0.00	1.0000
SI	48	0.2	0.00	1.0000
SI	64	0.2	0.00	1.0000
SI	80	0.2	0.00	1.0000
SI	96	0.2	0.00	1.0000
SI	128	0.2	0.00	1.0000
SI	32	0.2	0.01	0.9911
SI	48	0.2	0.01	0.9987
SI	64	0.2	0.01	0.9995
SI	80	0.2	0.01	0.9999
SI	96	0.2	0.01	1.0000
SI	128	0.2	0.01	1.0000
SI	32	0.2	0.02	0.9789
SI	48	0.2	0.02	0.9928
SI	64	0.2	0.02	0.9977
SI	80	0.2	0.02	0.9992
SI	96	0.2	0.02	0.9999
SI	128	0.2	0.02	1.0000
SI	32	0.2	0.05	0.9152
SI	48	0.2	0.05	0.9534
SI	64	0.2	0.05	0.9725
SI	80	0.2	0.05	0.9822
SI	96	0.2	0.05	0.9893
SI	128	0.2	0.05	0.9961
SI	32	0.2	0.10	0.7823
SI	48	0.2	0.10	0.8356
SI	64	0.2	0.10	0.8611
SI	80	0.2	0.10	0.8893
SI	96	0.2	0.10	0.9132
SI	128	0.2	0.10	0.9403
SI	32	0.2	0.25	0.5455
SI	48	0.2	0.25	0.5551
SI	64	0.2	0.25	0.5629
SI	80	0.2	0.25	0.5766
SI	96	0.2	0.25	0.5809
SI	128	0.2	0.25	0.5950
SI	32	0.3	0.00	1.0000
SI	48	0.3	0.00	1.0000
SI	64	0.3	0.00	1.0000
SI	80	0.3	0.00	1.0000
SI	96	0.3	0.00	1.0000
SI	128	0.3	0.00	1.0000
SI	32	0.3	0.01	0.9969
SI	48	0.3	0.01	0.9998
SI	64	0.3	0.01	1.0000
SI	80	0.3	0.01	1.0000
SI	96	0.3	0.01	1.0000
SI	128	0.3	0.01	1.0000
SI	32	0.3	0.02	0.9934
SI	48	0.3	0.02	0.9983
SI	64	0.3	0.02	0.9996
SI	80	0.3	0.02	1.0000
SI	96	0.3	0.02	1.0000
SI	128	0.3	0.02	1.0000

SI	32	0.3	0.05	0.9650
SI	48	0.3	0.05	0.9862
SI	64	0.3	0.05	0.9942
SI	80	0.3	0.05	0.9976
SI	96	0.3	0.05	0.9992
SI	128	0.3	0.05	0.9999
SI	32	0.3	0.10	0.8895
SI	48	0.3	0.10	0.9316
SI	64	0.3	0.10	0.9525
SI	80	0.3	0.10	0.9688
SI	96	0.3	0.10	0.9826
SI	128	0.3	0.10	0.9906
SI	32	0.3	0.25	0.6506
SI	48	0.3	0.25	0.6864
SI	64	0.3	0.25	0.7200
SI	80	0.3	0.25	0.7377
SI	96	0.3	0.25	0.7609
SI	128	0.3	0.25	0.7954
SI	32	0.4	0.00	1.0000
SI	48	0.4	0.00	1.0000
SI	64	0.4	0.00	1.0000
SI	80	0.4	0.00	1.0000
SI	96	0.4	0.00	1.0000
SI	128	0.4	0.00	1.0000
SI	32	0.4	0.01	0.9996
SI	48	0.4	0.01	1.0000
SI	64	0.4	0.01	1.0000
SI	80	0.4	0.01	1.0000
SI	96	0.4	0.01	1.0000
SI	128	0.4	0.01	1.0000
SI	32	0.4	0.02	0.9981
SI	48	0.4	0.02	0.9996
SI	64	0.4	0.02	1.0000
SI	80	0.4	0.02	1.0000
SI	96	0.4	0.02	1.0000
SI	128	0.4	0.02	1.0000
SI	32	0.4	0.05	0.9831
SI	48	0.4	0.05	0.9946
SI	64	0.4	0.05	0.9986
SI	80	0.4	0.05	0.9995
SI	96	0.4	0.05	0.9998
SI	128	0.4	0.05	1.0000
SI	32	0.4	0.10	0.9317
SI	48	0.4	0.10	0.9622
SI	64	0.4	0.10	0.9795
SI	80	0.4	0.10	0.9883
SI	96	0.4	0.10	0.9927
SI	128	0.4	0.10	0.9980
SI	32	0.4	0.25	0.7181
SI	48	0.4	0.25	0.7701
SI	64	0.4	0.25	0.8015
SI	80	0.4	0.25	0.8247
SI	96	0.4	0.25	0.8471
SI	128	0.4	0.25	0.8835
U	32	0.2	0.00	0.0000
U	48	0.2	0.00	0.0000

U	64	0.2	0.00	0.0000
U	80	0.2	0.00	0.0000
U	96	0.2	0.00	0.0000
U	128	0.2	0.00	0.0000
U	32	0.2	0.01	0.0000
U	48	0.2	0.01	0.0000
U	64	0.2	0.01	0.0000
U	80	0.2	0.01	0.0000
U	96	0.2	0.01	0.0000
U	128	0.2	0.01	0.0000
U	32	0.2	0.02	0.0000
U	48	0.2	0.02	0.0000
U	64	0.2	0.02	0.0000
U	80	0.2	0.02	0.0000
U	96	0.2	0.02	0.0000
U	128	0.2	0.02	0.0000
U	32	0.2	0.05	0.0001
U	48	0.2	0.05	0.0000
U	64	0.2	0.05	0.0000
U	80	0.2	0.05	0.0000
U	96	0.2	0.05	0.0000
U	128	0.2	0.05	0.0000
U	32	0.2	0.10	0.0001
U	48	0.2	0.10	0.0000
U	64	0.2	0.10	0.0000
U	80	0.2	0.10	0.0000
U	96	0.2	0.10	0.0000
U	128	0.2	0.10	0.0000
U	32	0.2	0.25	0.0139
U	48	0.2	0.25	0.0033
U	64	0.2	0.25	0.0007
U	80	0.2	0.25	0.0004
U	96	0.2	0.25	0.0001
U	128	0.2	0.25	0.0000
U	32	0.3	0.00	0.0000
U	48	0.3	0.00	0.0000
U	64	0.3	0.00	0.0000
U	80	0.3	0.00	0.0000
U	96	0.3	0.00	0.0000
U	128	0.3	0.00	0.0000
U	32	0.3	0.01	0.0000
U	48	0.3	0.01	0.0000
U	64	0.3	0.01	0.0000
U	80	0.3	0.01	0.0000
U	96	0.3	0.01	0.0000
U	128	0.3	0.01	0.0000
U	32	0.3	0.02	0.0000
U	48	0.3	0.02	0.0000
U	64	0.3	0.02	0.0000
U	80	0.3	0.02	0.0000
U	96	0.3	0.02	0.0000
U	128	0.3	0.02	0.0000
U	32	0.3	0.05	0.0000
U	48	0.3	0.05	0.0000
U	64	0.3	0.05	0.0000
U	80	0.3	0.05	0.0000

U	96	0.3	0.05	0.0000
U	128	0.3	0.05	0.0000
U	32	0.3	0.10	0.0000
U	48	0.3	0.10	0.0000
U	64	0.3	0.10	0.0000
U	80	0.3	0.10	0.0000
U	96	0.3	0.10	0.0000
U	128	0.3	0.10	0.0000
U	32	0.3	0.25	0.0083
U	48	0.3	0.25	0.0021
U	64	0.3	0.25	0.0005
U	80	0.3	0.25	0.0001
U	96	0.3	0.25	0.0000
U	128	0.3	0.25	0.0000
U	32	0.4	0.00	0.0000
U	48	0.4	0.00	0.0000
U	64	0.4	0.00	0.0000
U	80	0.4	0.00	0.0000
U	96	0.4	0.00	0.0000
U	128	0.4	0.00	0.0000
U	32	0.4	0.01	0.0000
U	48	0.4	0.01	0.0000
U	64	0.4	0.01	0.0000
U	80	0.4	0.01	0.0000
U	96	0.4	0.01	0.0000
U	128	0.4	0.01	0.0000
U	32	0.4	0.02	0.0000
U	48	0.4	0.02	0.0000
U	64	0.4	0.02	0.0000
U	80	0.4	0.02	0.0000
U	96	0.4	0.02	0.0000
U	128	0.4	0.02	0.0000
U	32	0.4	0.05	0.0000
U	48	0.4	0.05	0.0000
U	64	0.4	0.05	0.0000
U	80	0.4	0.05	0.0000
U	96	0.4	0.05	0.0000
U	128	0.4	0.05	0.0000
U	32	0.4	0.10	0.0000
U	48	0.4	0.10	0.0000
U	64	0.4	0.10	0.0000
U	80	0.4	0.10	0.0000
U	96	0.4	0.10	0.0000
U	128	0.4	0.10	0.0000
U	32	0.4	0.25	0.0082
U	48	0.4	0.25	0.0013
U	64	0.4	0.25	0.0003
U	80	0.4	0.25	0.0001
U	96	0.4	0.25	0.0000
U	128	0.4	0.25	0.0000

[a]Match call rates are per comparison of a pair of samples generated from a given relationship state. Genotyping error rates are per locus-level. Acronyms: Full Siblings, Minor Allele Frequency, Same Individual, Single Nucleotide Polymorphism, Unrelated.

Table S5.2 MSAT base case simulation results[a].

Relationship state	Number of loci	Number of alleles per locus	Allele frequency	ADO error rate	FA error rate	Match call rate
FS	5	5	0.20	0.00	0.00	0.012
FS	5	5	0.20	0.01	0.01	0.040
FS	5	5	0.20	0.05	0.02	0.058
FS	5	5	0.20	0.20	0.05	0.092
FS	5	10	0.10	0.00	0.00	0.002
FS	5	10	0.10	0.01	0.01	0.028
FS	5	10	0.10	0.05	0.02	0.040
FS	5	10	0.10	0.20	0.05	0.104
FS	5	20	0.05	0.00	0.00	0.000
FS	5	20	0.05	0.01	0.01	0.030
FS	5	20	0.05	0.05	0.02	0.022
FS	5	20	0.05	0.20	0.05	0.086
FS	10	5	0.00	0.00	0.00	0.000
FS	10	5	0.20	0.01	0.01	0.004
FS	10	5	0.20	0.05	0.02	0.007
FS	10	5	0.20	0.20	0.05	0.035
FS	10	10	0.10	0.00	0.00	0.000
FS	10	10	0.10	0.01	0.01	0.000
FS	10	10	0.10	0.05	0.02	0.004
FS	10	10	0.10	0.20	0.05	0.025
FS	10	20	0.05	0.00	0.00	0.000
FS	10	20	0.05	0.01	0.01	0.001
FS	10	20	0.05	0.05	0.02	0.006
FS	10	20	0.05	0.20	0.05	0.034
FS	15	5	0.20	0.00	0.00	0.000
FS	15	5	0.20	0.01	0.01	0.004
FS	15	5	0.20	0.05	0.02	0.004
FS	15	5	0.20	0.20	0.05	0.026
FS	15	10	0.10	0.00	0.00	0.000
FS	15	10	0.10	0.01	0.01	0.000
FS	15	10	0.10	0.05	0.02	0.002
FS	15	10	0.10	0.20	0.05	0.008
FS	15	20	0.05	0.00	0.00	0.000
FS	15	20	0.05	0.01	0.01	0.000
FS	15	20	0.05	0.05	0.02	0.000
FS	15	20	0.05	0.20	0.05	0.018
FS	20	5	0.20	0.00	0.00	0.000
FS	20	5	0.20	0.01	0.01	0.000
FS	20	5	0.20	0.05	0.02	0.001
FS	20	5	0.20	0.20	0.05	0.011
FS	20	10	0.10	0.00	0.00	0.000
FS	20	10	0.10	0.01	0.01	0.000
FS	20	10	0.10	0.05	0.02	0.000
FS	20	10	0.10	0.20	0.05	0.004
FS	20	20	0.05	0.00	0.00	0.000
FS	20	20	0.05	0.01	0.01	0.000
FS	20	20	0.05	0.05	0.02	0.000
FS	20	20	0.05	0.20	0.05	0.000
SI	5	5	0.00	0.00	0.01	1.000
SI	5	5	0.01	0.01	0.01	0.996
SI	5	5	0.05	0.02	0.01	0.950
SI	5	5	0.20	0.05	0.01	0.874

SI	5	10	0.00	0.00	0.01	1.000
SI	5	10	0.01	0.01	0.01	0.988
SI	5	10	0.05	0.02	0.01	0.984
SI	5	10	0.20	0.05	0.01	0.966
SI	5	20	0.00	0.00	0.01	1.000
SI	5	20	0.01	0.01	0.01	0.984
SI	5	20	0.05	0.02	0.01	0.976
SI	5	20	0.20	0.05	0.01	0.976
SI	10	5	0.20	0.00	0.00	1.000
SI	10	5	0.20	0.01	0.01	0.996
SI	10	5	0.20	0.05	0.02	0.988
SI	10	5	0.20	0.20	0.05	0.951
SI	10	10	0.10	0.00	0.00	1.000
SI	10	10	0.10	0.01	0.01	0.997
SI	10	10	0.10	0.05	0.02	0.993
SI	10	10	0.10	0.20	0.05	0.985
SI	10	20	0.05	0.00	0.00	1.000
SI	10	20	0.05	0.01	0.01	0.997
SI	10	20	0.05	0.05	0.02	0.998
SI	10	20	0.05	0.20	0.05	1.000
SI	15	5	0.20	0.00	0.00	1.000
SI	15	5	0.20	0.01	0.01	0.998
SI	15	5	0.20	0.05	0.02	0.997
SI	15	5	0.20	0.20	0.05	0.980
SI	15	10	0.10	0.00	0.00	1.000
SI	15	10	0.10	0.01	0.01	1.000
SI	15	10	0.10	0.05	0.02	0.998
SI	15	10	0.10	0.20	0.05	0.997
SI	15	20	0.05	0.00	0.00	1.000
SI	15	20	0.05	0.01	0.01	1.000
SI	15	20	0.05	0.05	0.02	1.000
SI	15	20	0.05	0.20	0.05	1.000
SI	20	5	0.20	0.00	0.00	1.000
SI	20	5	0.20	0.01	0.01	1.000
SI	20	5	0.20	0.05	0.02	0.999
SI	20	5	0.20	0.20	0.05	0.996
SI	20	10	0.10	0.00	0.00	1.000
SI	20	10	0.10	0.01	0.01	1.000
SI	20	10	0.10	0.05	0.02	1.000
SI	20	10	0.10	0.20	0.05	0.998
SI	20	20	0.05	0.00	0.00	1.000
SI	20	20	0.05	0.01	0.01	1.000
SI	20	20	0.05	0.05	0.02	1.000
SI	20	20	0.05	0.20	0.05	1.000
U	5	5	0.20	0.00	0.00	0.000
U	5	5	0.20	0.01	0.01	0.000
U	5	5	0.20	0.05	0.02	0.000
U	5	5	0.20	0.20	0.05	0.000
U	5	10	0.10	0.00	0.00	0.000
U	5	10	0.10	0.01	0.01	0.000
U	5	10	0.10	0.05	0.02	0.000
U	5	10	0.10	0.20	0.05	0.000
U	5	20	0.05	0.00	0.00	0.000
U	5	20	0.05	0.01	0.01	0.000
U	5	20	0.05	0.05	0.02	0.000
U	5	20	0.05	0.20	0.05	0.000

U	10	5	0.20	0.00	0.00	0.000
U	10	5	0.20	0.01	0.01	0.000
U	10	5	0.20	0.05	0.02	0.000
U	10	5	0.20	0.20	0.05	0.000
U	10	10	0.10	0.00	0.00	0.000
U	10	10	0.10	0.01	0.01	0.000
U	10	10	0.10	0.05	0.02	0.000
U	10	10	0.10	0.20	0.05	0.000
U	10	20	0.05	0.00	0.00	0.000
U	10	20	0.05	0.01	0.01	0.000
U	10	20	0.05	0.05	0.02	0.000
U	10	20	0.05	0.20	0.05	0.000
U	15	5	0.20	0.00	0.00	0.000
U	15	5	0.20	0.01	0.01	0.000
U	15	5	0.20	0.05	0.02	0.000
U	15	5	0.20	0.20	0.05	0.000
U	15	10	0.10	0.00	0.00	0.000
U	15	10	0.10	0.01	0.01	0.000
U	15	10	0.10	0.05	0.02	0.000
U	15	10	0.10	0.20	0.05	0.000
U	15	20	0.05	0.00	0.00	0.000
U	15	20	0.05	0.01	0.01	0.000
U	15	20	0.05	0.05	0.02	0.000
U	15	20	0.05	0.20	0.05	0.000
U	20	5	0.20	0.00	0.00	0.000
U	20	5	0.20	0.01	0.01	0.000
U	20	5	0.20	0.05	0.02	0.000
U	20	5	0.20	0.20	0.05	0.000
U	20	10	0.10	0.00	0.00	0.000
U	20	10	0.10	0.01	0.01	0.000
U	20	10	0.10	0.05	0.02	0.000
U	20	10	0.10	0.20	0.05	0.000
U	20	20	0.05	0.00	0.00	0.000
U	20	20	0.05	0.01	0.01	0.000
U	20	20	0.05	0.05	0.02	0.000
U	20	20	0.05	0.20	0.05	0.000

[a] Match call rates are per comparison of a pair of samples generated from a given relationship state. Genotyping error rates are per locus-level. Acronyms: Allelic Drop Out, False Allele, Full Siblings, MicroSATellite, Same Individual, Unrelated.
